# Harnessing defective interfering particles and lipid nanoparticles for effective delivery of an anti-dengue virus RNA therapy

**DOI:** 10.1016/j.omtn.2024.102424

**Published:** 2024-12-12

**Authors:** Min-Hsuan Lin, Pramila Maniam, Dongsheng Li, Bing Tang, Cameron R. Bishop, Andreas Suhrbier, Lucy Wales- Earl, Yaman Tayyar, Nigel A.J. McMillan, Li Li, David Harrich

**Affiliations:** 1 Program of Infection and Inflammation, QIMR Berghofer Medical Research Institute, Herston, QLD 4006, Australia; 2 Global Virus Network (GVN) Center of Excellence, Australian Infectious Disease Research Centre, Brisbane, QLD 4072, Australia; 3 Menzies Health Institute Queensland and School of Pharmacy and Medical Science, Griffith University, Gold Coast, QLD 4222, Australia; 4 Prorenata Biotech, Molendinar, QLD 4214, Australia; 5 Australian Institute for Bioengineering and Nanotechnology, the University of Queensland, St Lucia, QLD 4072, Australia

**Keywords:** MT: Delivery Strategies, defective, interfering, RNA, DIPs, LNPs, dengue, therapeutic, antiviral, mouse

## Abstract

Currently, no approved antiviral drugs target dengue virus (DENV) infection, leaving treatment reliant on supportive care. DENV vaccine efficacy varies depending on the vaccine type, the circulating serotype, and vaccine coverage. We investigated defective interfering particles (DIPs) and lipid nanoparticles (LNPs) to deliver DI290, an anti-DENV DI RNA. Both DIPs and DI290-loaded LNPs (LNP-290) effectively suppressed DENV infection in human primary monocyte-derived macrophages (MDMs), THP-1 macrophages, and fibroblasts-natural DENV targets. Inhibiting interferon (IFN) signaling with a Janus kinase 1/2 inhibitor or an IFN-α/β receptor 1 (IFNAR1)-binding antibody blocked DIP and LNP-290 antiviral activity. LNP-290 demonstrated a greater than log10 inhibition of DENV viral loads in IFNAR-deficient (*Ifnar*^*−/−*^) and IFN regulatory factor (IRF) 3 and 7 double knockout (*Irf3/7*^*−/−*^) mice. Pathway analysis of RNA sequencing data from LNP-treated C57BL/6J mice, *Ifnar*^*−/−*^ mice, and human MDMs treated with LNPs or DENV DIPs indicated DI290 treatment enhanced IFN responses, suggesting IFN-λ and IFN-γ provided antiviral activity when IFN-α/β responses were diminished. While viral interference by DI290 is possible, results did not support RNA replication competition as an inhibition mechanism. These findings suggest that DI290 may be a promising DENV therapeutic by activating the innate immune system.

## Graphical abstract

**Figure:**
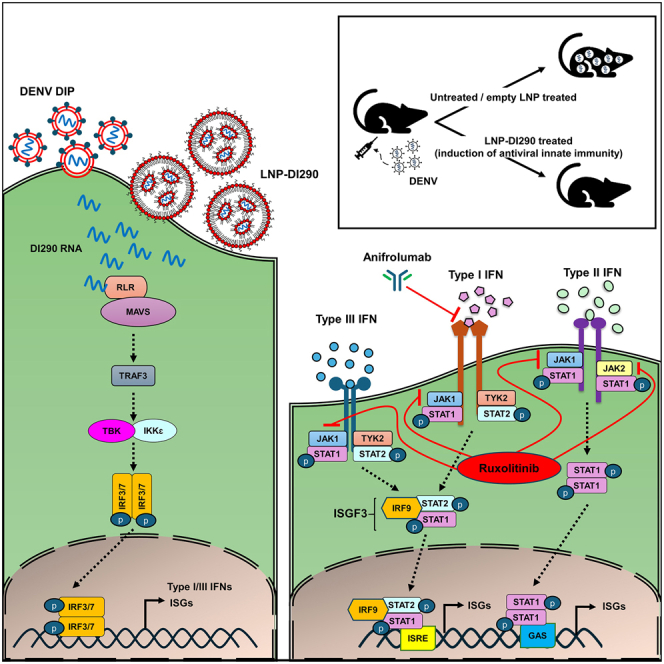

## Teaser

Harrich and colleagues demonstrate that DIPs delivered via LNPs provide a potent RNA-based antiviral strategy against DENV. This innovative approach showcases the therapeutic potential of DIPs/LNPs, offering a targeted, efficient delivery mechanism that enhances treatment efficacy against viral infections for broader application.

## Introduction

Dengue virus (DENV) is a single-stranded RNA virus from the *Flaviviridae* family transmitted to humans by the *Aedes aegypti* mosquito. It is estimated that approximately 390 million people are infected with DENV each year, making it one of the most common mosquito-borne viral diseases worldwide.^1^ The disease can manifest with a wide range of symptoms, from mild flu-like illness to severe and potentially fatal forms such as dengue hemorrhagic fever and dengue shock syndrome. The development of antiviral drugs for DENV is crucial; they can help to reduce the severity of the infection and prevent complications. Traditional antiviral drugs typically function by directly targeting the virus, either by impeding its replication or by obstructing its entry into and infection of host cells. Nonetheless, because of the absence of approved specific antiviral treatments and restricted availability of vaccines for DENV,^2^^,^^3^ disease management primarily hinges on mosquito control measures, prompt detection, and supportive care of patients to ameliorate fever, dehydration, bleeding, and shock.

We described herein DI290 RNA, a short 290-nucleotide defective interfering (DI) RNA derived from DENV.^4^^,^^5^^,^^6^^,^^7^ DI RNAs are produced by RNA viruses due to errors in the viral RNA replication process, reviewed in Vignuzzi and López and Genoyer and López.^8^^,^^9^ These errors lead to incomplete or shortened copies of the viral genome, which lack some or all of the genes necessary for viral replication. DI290 RNA retains the full 5′ and 3′ untranslated regions (UTRs) of the viral genome, but it lacks all open reading frames. DI RNAs, including DI290 RNA, can disrupt the replication of the parent virus through various mechanisms.^8^^,^^10^ DI RNAs are suggested to compete with the parent virus for limited resources within the host cell (viral interference), such as enzymes and nucleotides essential for viral RNA replication and packaging of newly synthesized viral RNA into new virions. We previously showed that Vero cells expressing DENV structural (S) and non-structural (NS) proteins through separate lentiviral vectors can replicate DI290 RNA, suggesting that DI290 RNA has the capacity to compete with viral genomic RNA for replication in virus-infected cells.^5^ Through these mechanisms, DI RNAs can diminish the virus’s capacity to propagate and induce disease. This has been demonstrated in studies of many different viruses including influenza A,^11^^,^^12^^,^^13^ Zika virus,^14^ chikungunya virus,^15^ severe acute respiratory syndrome coronavirus 2,^16^^,^^17^ and HIV-1.^18^

We used stable cell lines to synthetically generate DENV-based DIPs, which were purified and concentrated.^6^ Our findings demonstrated that DENV-based DIPs exhibit broad-spectrum antiviral activity by eliciting innate immune responses in cells.^6^ DIP and DI RNA broad-spectrum antiviral activity elicited via IFN responses has been reported by others.^15^^,^^19^^,^^20^^,^^21^^,^^22^^,^^23^ There is evidence indicating that DI RNAs bind the retinoic acid-inducible gene-I (RIG-I)-like receptors (RLRs), RIG-I and melanoma differentiation-associated protein 5 (MDA5), activating this signaling pathway and initiating antiviral responses in host cells.^24^^,^^25^ A direct interaction between the DENV 5′ UTR and RIG-I has been reported.^26^ Upon recognition of a target RNA, RLRs undergo a conformational change, exposing its caspase activation and recruitment domains (CARDs). These exposed CARDs of RLRs then interact with the mitochondrial antiviral signaling protein (MAVS) located on the outer mitochondrial membrane. This interaction initiates a signaling cascade involving the activation of various downstream molecules, including TANK-binding kinase 1 (TBK1) and I-kappa-B kinase epsilon (IKKε). MAVS deficiency in cells abrogates the ability of DI RNA to induce antiviral responses to DI RNAs.^27^ TBK1 and IKKε subsequently phosphorylate IFN regulatory factor (IRF)3 and IRF7, while nuclear factor (NF)-κB activation involves the IKK complex (IKKα/β/γ). Phosphorylated IRF3, IRF7 and liberated NF-κB translocate to the nucleus and induce the transcription of type I/III IFNs and other proinflammatory cytokines, reviewed in.^28^ DI RNAs originating from various RNA viruses have been shown to activate innate immunity and promote an antiviral response in host cells.^23^^,^^27^^,^^29^^,^^30^^,^^31^ This then leads to the inhibition of viral replication and dissemination.

Herein we explored DI290’s capacity to impede DENV replication in primary human macrophages, foreskin-derived fibroblasts, and in two mouse models of DENV-2 infection. Our findings indicate that DI290 shows promise as an anti-DENV agent by activating IFN responses, capable of diminishing virus replication and decreasing viral titers *in vivo*. These findings illustrate DI290’s potential as a therapeutic intervention against DENV infection.

## Results

### DI290 RNA protects human macrophage and fibroblasts from DENV infection

**Figure 1:**
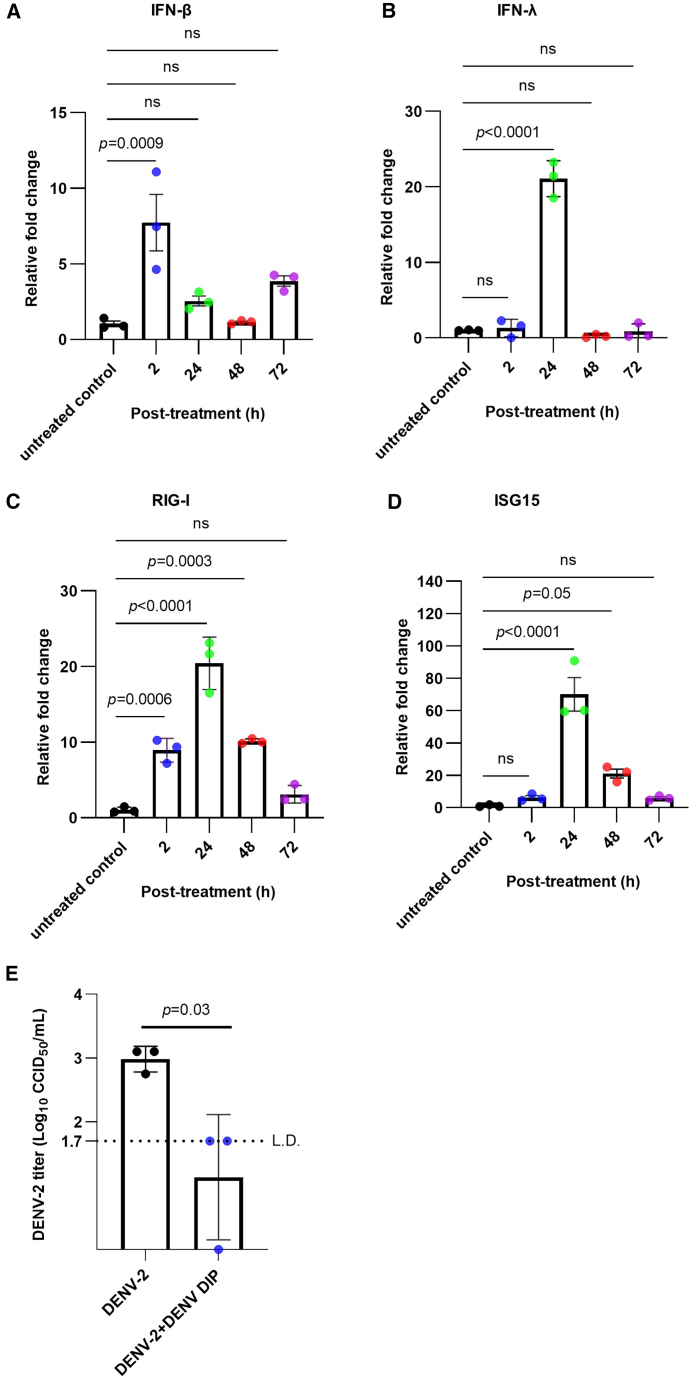
DENV DIPs stimulate host innate immune responses in human primary MDMs and inhibit DENV-2 replication (A–D) Monocytes were isolated from human PBMCs using CD14-positive selection and maturated to MDMs with M-CSF and GM-CSF for 5 days. MDMs were then treated with DENV DIPs (at a dosage equivalent to 1,000 DI290 RNA copies [=0.2 fg] per cell) for 2, 24, 48, and 72 h. Total RNA was extracted from the cells and the levels of IFN-β, IFN-λ, RIG-I, and ISG15 mRNA were quantified by RT-qPCR. The fold change relative to the untreated control cells was calculated (*n* = 3 per group). The data are shown as the mean ± SD. Statistical analysis was performed by one-way ANOVA. (E) MDMs were infected with DENV-2 (MOI = 1 CCID_50_ per cell). After 3 h, the cells were washed with 1× PBS and incubated with culture medium containing DENV DIP (at a dosage equivalent to 1,000 DI290 RNA copies [0.2 fg] per cell). DENV-2 titers in culture supernatant were measured by CCID_50_ assay at 3 days post-infection (*n* = 3 per group). The data are shown as the mean ± SD. Statistical analysis was performed by Student’s t test. L.D., limit of detection; ns, not significant.

**Figure 2:**
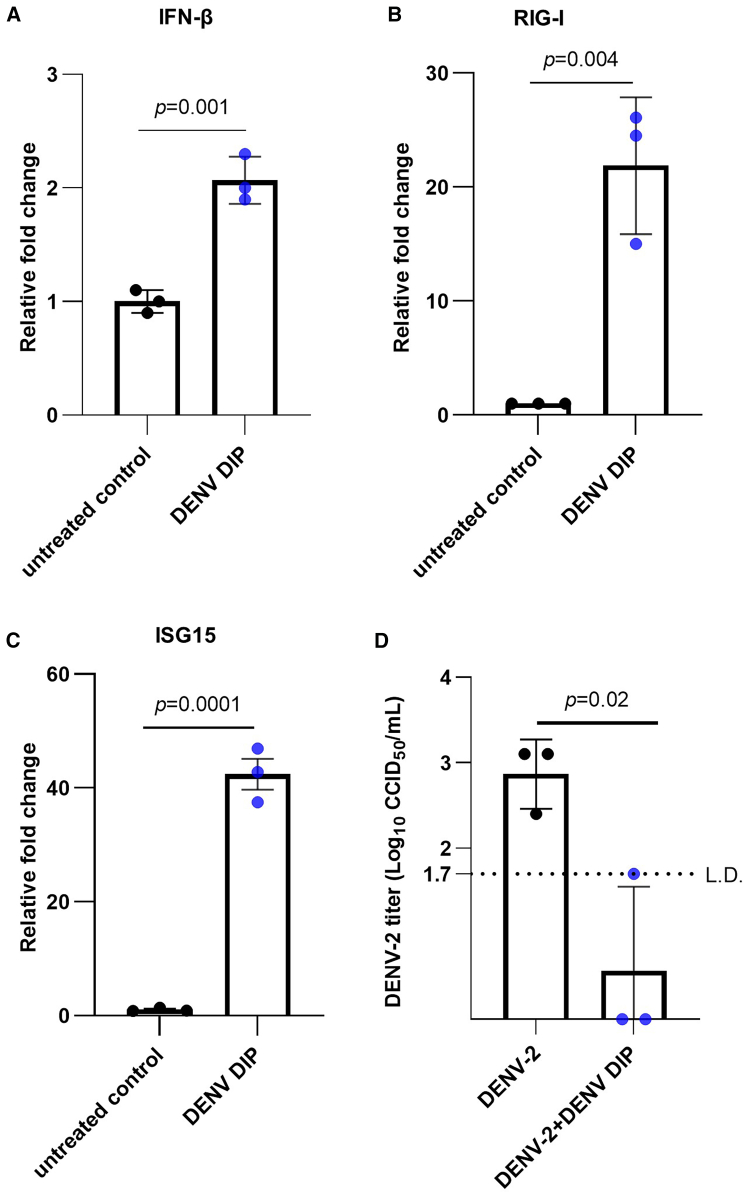
DENV DIPs activate IFN responses and inhibits DENV-2 in HFF-1 human fibroblast cells (A-C) HFF-1 cells were treated with DENV DIP (at a dosage equivalent to 1,000 DI290 RNA copies [=0.2 fg] per cell) for 24 h. Total RNA was extracted from the cells and the levels of IFN-β, RIG-I, and ISG15 mRNA were quantified by RT-qPCR. The fold change relative to the untreated control cells was calculated (*n* = 3 per group). Data are shown as the mean ± SD. Statistical analysis was performed by Student’s t test. (D) HFF-1 cells were infected with DENV-2 (MOI = 0.1 CCID_50_/cell). After 3 h, the cells were washed with 1× PBS and incubated with culture medium containing DENV DIP (at a dosage equivalent to 1,000 DI290 RNA copies [=0.2 fg] per cell). DENV-2 titers in culture supernatant were measured by CCID_50_ assay at 3 days post-infection (*n* = 3 per group). Data are shown as the mean ± SD. Statistical analysis was performed by Student’s t test. L.D., limit of detection.

Transmission of DENV by mosquitoes through skin bites involves several dermal and epidermal cells that are susceptible to infection, including keratinocytes, dendritic cells, Langerhans cells, and fibroblast cells.^32^^,^^33^^,^^34^ After entering the bloodstream, DENV predominantly infects monocytes and macrophages, which act as a cellular reservoir for virus replication.^35^^,^^36^^,^^37^^,^^38^^,^^39^ In this study, our first objective was to examine whether DENV DIPs induce IFN responses in human primary monocyte-derived macrophages (MDMs), similar to observations made previously in Huh7 hepatoma cells.^6^ MDMs treated with DENV DIPs exhibited a transient and rapid increase in IFN-β mRNA levels at 2 h aftertreatment, alongside transiently elevated levels of IFN-λ mRNA at 24 h (Figures 1A and 1B, respectively). Moreover, RIG-I showed significant upregulation at 2 h after treatment (Figure 1C), while both RIG-I and IFN-stimulated gene 15 (ISG15) were upregulated at subsequent time points (Figures 1C and 1D, respectively). Similar observations were made in THP-1 macrophage cells treated with DIPs, demonstrating notable upregulation of RIG-I and ISG15 (Figures S1A and S1B). In addition, HFF-1 cells, a human fibroblast cell line, exhibited an approximately 2-fold increase in IFN-β mRNA levels and a 20- to 40-fold increase in RIG-I and ISG15 mRNA levels, respectively, at 24 h after DIP treatment (Figures 2A–2D). ELISAs of the supernatant and cell lysates confirmed upregulated levels of IFN-β and ISG15 in both infected and uninfected HFF-1 cells treated with DENV DIPs, while untreated infected HFF-1 cells did not show increased IFN-β and ISG15 levels (Figures S2 and S3). This pattern of IFN-β expression was observed previously in Huh7 cells.^6^

All three cell types were infected with DENV-2 for 3 h, using a multiplicity of infection (MOI) of 0.1 for HFF-1 cells and a MOI of 1.0 for MDMs and THP-1. After removing the DENV inoculum, the cells were treated with DENV DIPs, delivering 1,000 molecules of DI290 RNA per cell or with DIP storage buffer as a negative control. The viral titers in the supernatant from control-treated cells were approximately 10^3^ CCID_50_/mL. However, viral titers in DENV DIP-treated MDM, THP-1 and HFF-1 cells were either at the limit of detection or below (Figures 1E, 2D, and S1C), indicating that DENV DIPs effectively inhibit DENV-2 replication *in vitro* in macrophages and fibroblasts, consistent with previous observations in other cell types.^5^^,^^6^ In summary, these findings demonstrate that DENV DIPs stimulate cellular innate immune responses and have potent antiviral activity in human MDM, THP-1, and fibroblast cells.

### LNPs delivering DI290 RNA inhibit DENV-2 *in vitro* and requires IFN signaling pathways in HFF-1 cells

**Figure 3:**
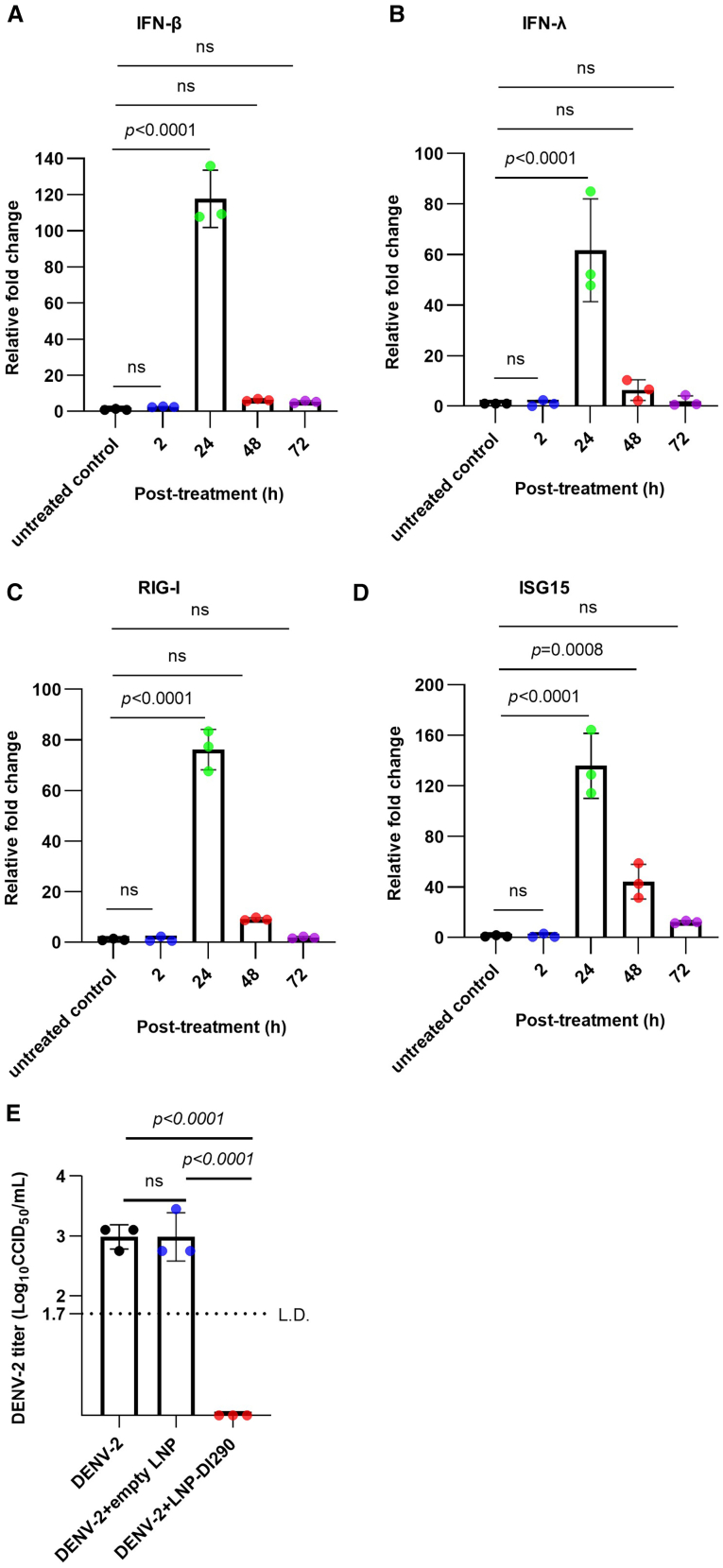
LNP-DI290 stimulates host innate immune responses in human primary MDMs and inhibits DENV-2 replication (A–D) MDMs were treated with LNP-DI290 (at a dosage equivalent to 25 pg DI290 RNA per cell) for 2, 24, 48, and 72 h. Total RNA was extracted from the cells and the levels of IFN-β, IFN-λ, RIG-I, and ISG15 mRNA were quantified by RT-qPCR. The fold change relative to the untreated control cells was calculated (*n* = 3 per group). Data are shown as the mean ± SD. Statistical analysis was performed by one-way ANOVA. (E) MDM were infected with DENV-2 (MOI = 1 CCID_50_ per cell). After 3 h, the cells were washed with 1× PBS and incubated with either culture medium alone, medium containing either empty LNP or LNP-DI290 (at a dosage equivalent to 25 pg DI290 RNA per cell). DENV-2 titers in culture supernatant were measured by CCID_50_ assay at 3 days post-infection (*n* = 3 per group). Data are shown as the mean ± SD. Statistical analysis was performed by one-way ANOVA. L.D., limit of detection; ns, not significant.

**Figure 4:**
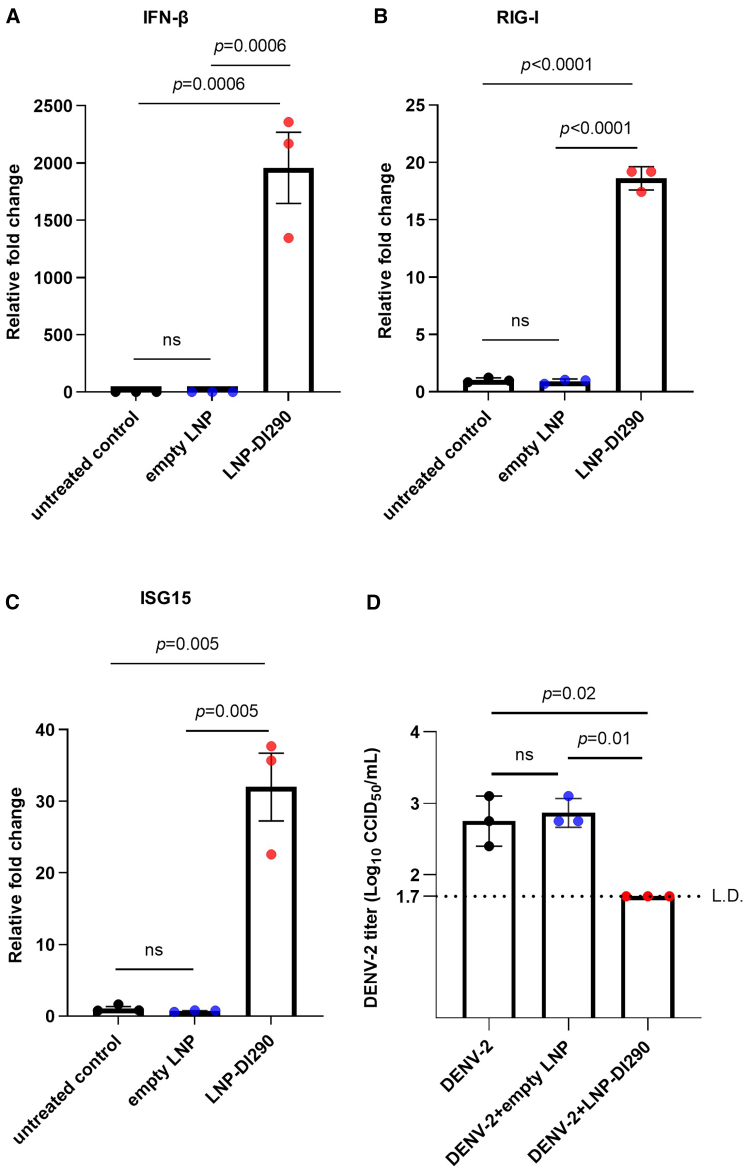
LNP-DI290 stimulates host innate immune responses in HFF-1 cells and inhibits DENV-2 replication (A–C) HFF-1 cells were treated with empty LNP or LNP-DI290 (at a dosage equivalent to 25 pg DI290 RNA per cell) for 24 h. Total RNA was extracted from the cells and the levels of IFN-β, RIG-I, and ISG15 mRNA were quantified by RT-qPCR. The fold change relative to the untreated control cells was calculated (*n* = 3 per group). Data are shown as the mean ± SD. Statistical analysis was performed by one-way ANOVA. (D) HFF-1 cells were infected with DENV-2 (MOI = 0.1 CCID_50_ per cell). After 3 h, the cells were washed with 1× PBS and incubated with either culture medium alone, medium containing empty LNP or LNP-DI290 (at a dosage equivalent to 25 pg DI290 RNA per cell). DENV-2 titers in culture supernatant were measured by CCID_50_ assay at 3 days post-infection (*n* = 3 per group). Data are shown as the mean ± SD. Statistical analysis was performed by one-way ANOVA. L.D., limit of detection; ns, not significant.

Several groups reported successful *in vitro* and *in vivo* delivery of DI RNA/DVGs using LNPs.^16^^,^^17^^,^^23^ As an established method for delivery of DI RNA, LNPs delivering DI290 RNA were used to treat MDM and HFF-1 cells *in vitro* to recapitulate the stimulation of IFN responses,^40^ that was observed with DIP treatment. MDMs and HFF-1 cells were treated with LNP-DI290 delivering 250 ng of DI290 for 24 h. RT-qPCR analyses for RNA from treated and untreated cells showed that LNP-DI290 significantly upregulated IFN-β, IFN-λ, RIG-I, and ISG15 mRNA levels in MDMs and HFF-1 cells compared with cells treated with empty LNPs and untreated cells after 24 h (Figures 3A–3D, 4A–4C, and S4A–S4D). These results demonstrate that LNPs effectively deliver DI290 RNA, stimulating innate immune responses and upregulating IFNs and ISGs in MDMs and HFF-1 cells.

MDMs and HFF-1 cells were also infected with DENV-2 and were then treated as above with LNP-290. As shown in Figures 3E and 4D, cells treated with LNP-290 exhibited strong inhibition of DENV-2 compared with cells treated with empty LNPs and untreated cells.

Ruxolitinib (Rux) is a potent, reversible, and selective Janus kinase (JAK)1 and JAK2 inhibitor.^41^ JAK1 and tyrosine kinase 2 (TYK2) mediate type I/III IFNs signaling, while JAK1 and JAK2 mediate type II IFN signaling. When IFNs bind to their respective receptors on the cell surface such as the IFNAR for type I IFN, they initiate a signaling cascade that leads to the activation of ISGs, which mediate the antiviral responses. In type I/III IFN signaling, the binding of IFNs to their receptors activates JAK1 and TYK2, leading to the phosphorylation of signal transducer and activator of transcription (STAT) proteins, mainly STAT1 and STAT2. These STATs then form the ISG factor 3 transcription complex with IRF9, which drives ISG expression. In contrast, type II IFN signaling activates JAK1 and JAK2, resulting in STAT1 phosphorylation and activation, which also contributes to the ISG activation. We investigated whether the JAK-STAT signaling pathway is involved in DI290-mediated innate immune response using Rux. HFF-1 cells were incubated with medium containing 5, 10, and 20 μM Rux for up to 72 h. An MTS assay showed that the cell proliferation profiles of DMSO-treated HFF-1 cells and those treated with 5 μM Rux were not significantly different (Figure S5A). HFF-1 cells then were incubated with culture medium containing 5 μM Rux for 1 h and RT-qPCR analysis of total cellular RNA collected at 24 h after treatment showed that ISG15 expression levels were significantly downregulated in Rux-treated cells compared with HFF-1 cells treated with LNP-290 (Figure S5B). In DENV-2 infected cells, while LNP-290 treatment alone significantly inhibited virus production, Rux restored viral levels to those observed in DMSO-treated cells (Figure S5C). These results support a role for the JAK-STAT signaling and ISG gene expression pathways in the inhibition of DENV-2 replication by LNP-290 in HFF-1 cells.

Next, HFF-1 cells were infected with DENV-2 and treated with anifrolumab or with an isotype matched negative control antibody. Anifrolumab is a monoclonal antibody that targets the IFN-α/β receptor 1 (IFNAR1), blocking the receptor’s ability to bind type I IFNs. By binding IFNAR1, anifrolumab prevents the activation of the JAK-STAT signaling pathway by type I IFNs.^42^ As shown in Figure S6A, anifrolumab inhibited ISG15 mRNA levels in HFF-1 cells treated with LNP-290. In addition, viral levels in culture supernatants were measured at 72 h post infection (h.p.i.). The results indicate that blocking IFNAR1 with anifrolumab significantly diminished the antiviral effects of LNP-290 in HFF-1 cells (Figure S6B). Moreover, we found that the addition of Rux or anifrolumab to HFF-1 cell also blocked ISG15 activation by DIPs demonstrating that LNP-290 and DIPs activate INF responses similarly (Figure S7). Taken together, the findings from experiments using Rux and anifrolumab in DENV-infected HFF-1 cells demonstrate that IFN signaling plays a crucial role in the antiviral activity induced by LNP-290 and DIPs.

### LNPs delivering DI290 RNA inhibit DENV-2 *in vivo*

**Figure 5:**
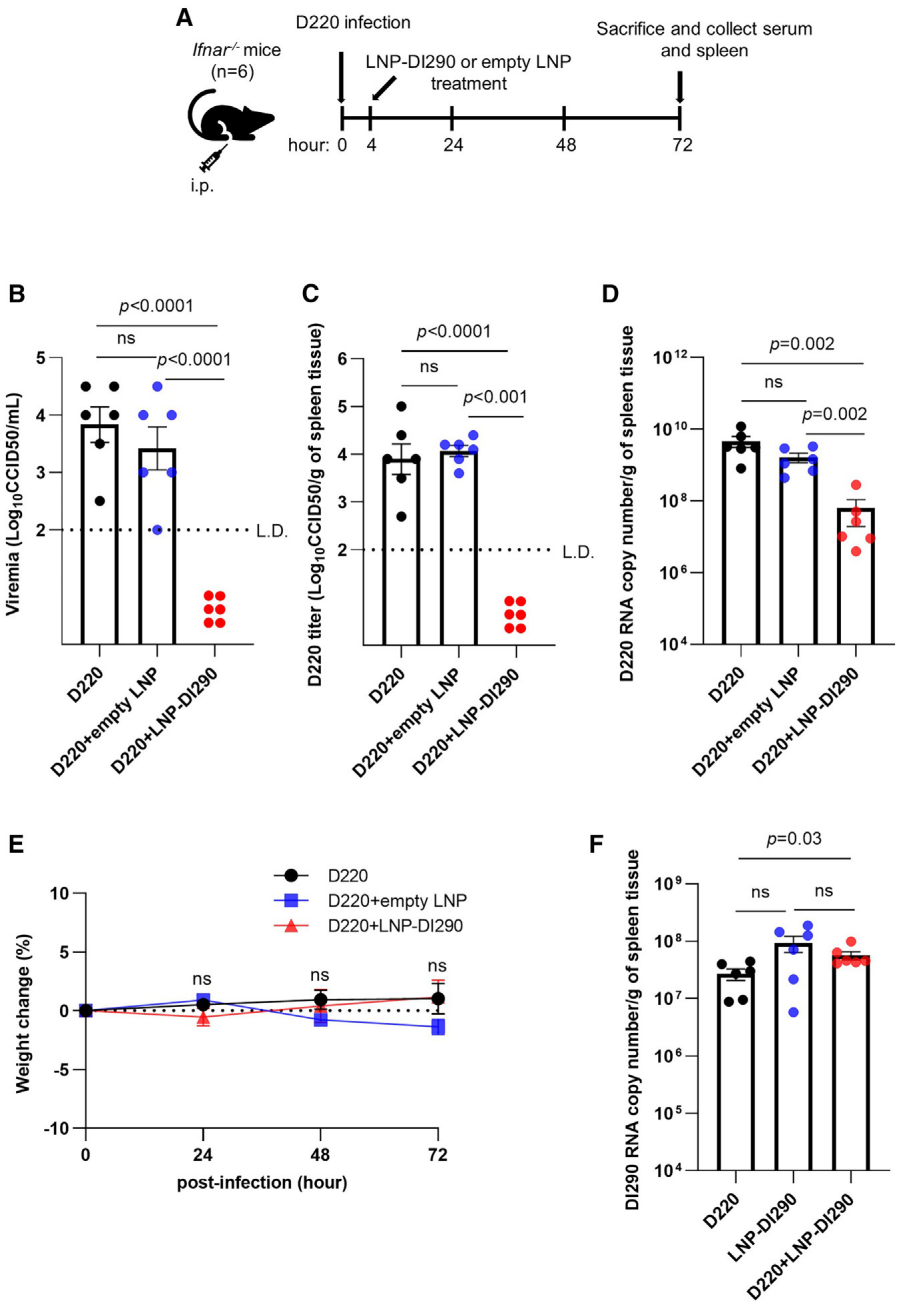
LNP-DI290 inhibits DENV replication in infected *Ifnar*^*−/−*^ mice (A) Schematic representation of the experimental design. IFNAR-deficient mice were i.p. infected with mouse-adapted DENV-2 strain D220 (10^4^ CCID_50_ per mouse). After 4 h infection, the mice were i.p. treated with either empty LNP or LNP-DI290 (15 μg DI290 RNA per mouse). (B and C) Viremia and virus titers in spleen were measured by CCID_50_ assay at 72 h post-infection (*n* = 6 per group). Data are shown as the mean ± SEM. Statistical analysis was performed by one-way ANOVA. (D) The level of DENV-2 D220 RNA in spleen was measured by RT-qPCR using primers for the D220 NS5 region (*n* = 6 per group). Data are shown as the mean ± SEM. Statistical analysis was performed by Kolmogorov-Smirnov exact test. (E) Mean percent body weight change related to each mouse’s weight on day 0 (*n* = 6 per group). Data are shown as the mean ± SEM. Statistical analysis was performed by one-way ANOVA. Significance was not reached on any day. (F) The level of DI290 RNA in spleen samples was measured by RT-qPCR (*n* = 6 per group). Data are shown as the mean ± SEM. Statistical analysis was performed by Kolmogorov-Smirnov exact test. L.D., limit of detection; ns, not significant.

LNPs have the capability to deliver significant amounts of RNA *in vivo*, which prompted an evaluation of the antiviral efficacy of LNP-DI290 in a DENV-mouse model. *Ifnar*^*−/−*^ mice are genetically modified to have a specific deletion of the gene encoding the IFNAR1.^43^ By knocking out the *Ifnar1* gene, these mice lack the receptor necessary for cells to respond to type I IFNs, thereby exhibiting heightened susceptibility to infection by viruses, including DENV.^44^ These mice retain type II IFN and type III IFN responses. *Ifnar*^*−/−*^ mice were infected by intraperitoneal (i.p.) injection of mouse-adapted D220 DENV-2 strain (10^4^ CCID_50_ per mouse), followed by treatment with LNP-DI290 delivering 15 μg of RNA, empty LNPs, or buffer only at 4 h.p.i. (Figure 5A). Serum and spleen samples were collected at 72 h.p.i. and subjected to assays for infectious virus or viral RNA. Infectious D220 was detected in the serum and spleen at more than 3 log_10_ CCID_50_/mL, while infectious virus was undetectable in the spleen and serum samples from mice treated with LNP-DI290 (Figures 5B and 5C). In contrast, all samples from untreated and empty LNP-treated mice exhibited measurable titers of D220, illustrating the antiviral activity of LNP-DI290 *in vivo*.

RT-qPCR analysis of total RNA from spleen samples of all mice revealed a significant reduction in viral RNA in LNP-DI290-treated mice compared with those treated with empty LNPs or buffer (Figure 5D). This indicated that while the LNP-DI290-treated mice were infected with DENV-2, the infection did not lead to measurable levels of viremia *in vivo*. In addition, LNP-DI290-treated mice did not experience significant weight loss (Figure 5E), suggesting that the treatment neither exacerbated DENV pathogenesis nor caused unexpected adverse effects during the experiment. Overall, the data demonstrate that LNP-DI290 delivering 15 μg of DI290 RNA effectively inhibited D220 replication and reduced viremia in *Ifnar*^*−/−*^ mice without observable adverse effects.

We next investigated whether viral interference might be a mechanism of DENV inhibition by measuring the levels of DI290 in spleen samples. RT-qPCR analysis of D220 virus stocks did not detect measurable levels of DI290 (Figure S8A), while DENV genomic RNA exceeded 10^9^ copies/mL (Figure S8B). In untreated, DENV-infected *Ifnar*^*−/−*^ mice, DI290 was detected at approximately 3 × 10^7^ RNA copies/mL (Figure 5F), resulting in a DI290-to-viral genomic RNA ratio of approximately 1:170. In contrast, in DENV-infected mice treated with LNP-290, the DI290-to-viral RNA ratio increased to approximately 1:1 (Figure 5F). Surprisingly, uninfected mice treated with LNP-290 exhibited comparable DI290 levels (Figure 5F). However, these results do not support the hypothesis that DI290 undergoes robust replication in DENV-infected cells or that replication interference is a dominant mechanism of action, such as by DI290 RNA replication.

### LNP-DI290 can protect mice from DENV infection independent of IRF3/7

**Figure 6:**
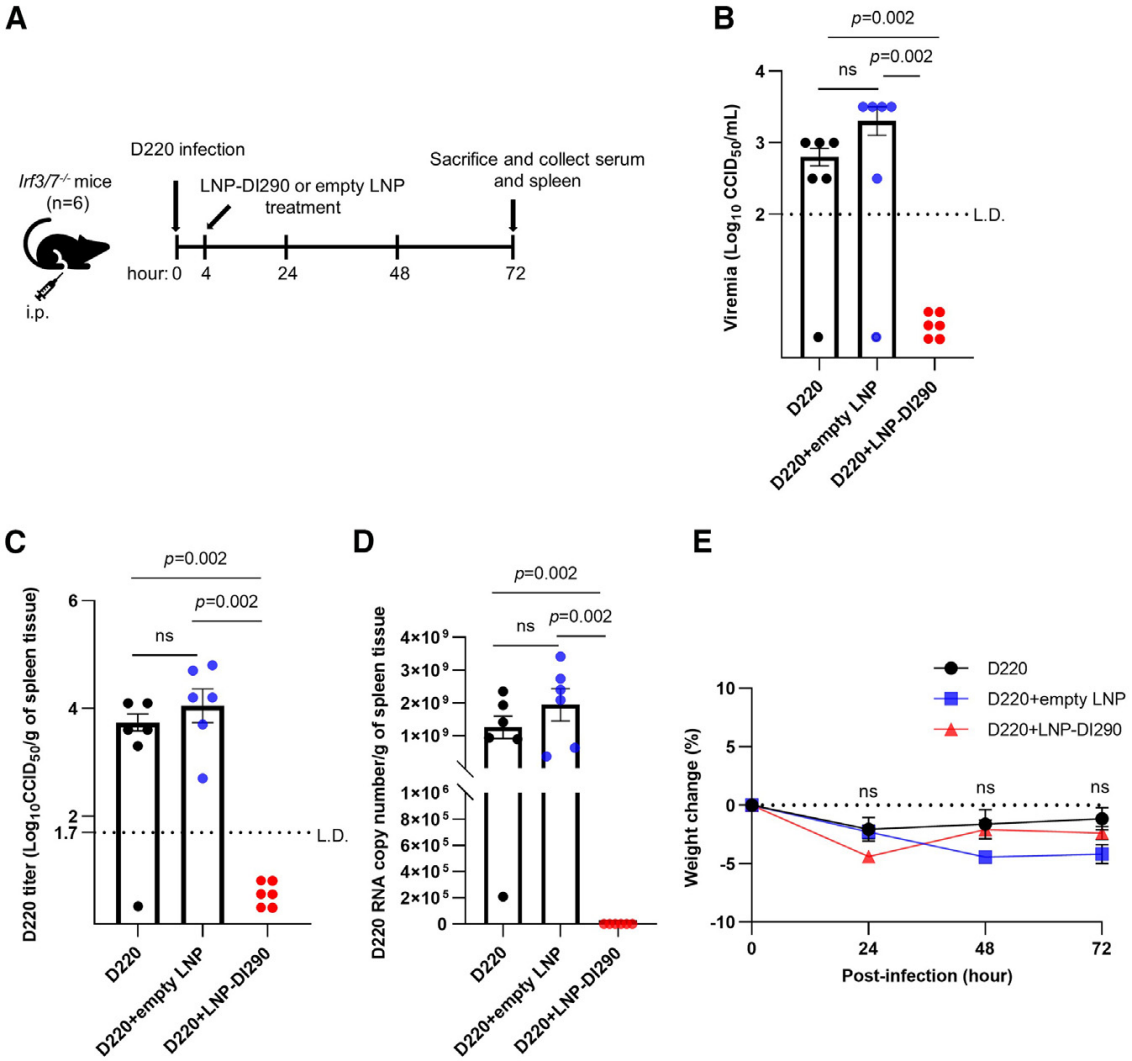
LNP-DI290 block DENV replication in infected *Irf3/7*^*−/−*^ mice (A) Schematic representation of the experimental design. IRF3/IRF7 DKO mice were *i.p*. infected with D220 (10^4^ CCID_50_ per mouse). After 4 h infection, the mice were i.p. treated with either empty LNP or LNP-DI290 (15 μg DI290 RNA per mouse). (B and C) Viremia and virus titers in spleen were measured by CCID_50_ assay at 72 h post-infection (*n* = 6 per group). Data are shown as the mean ± SEM. Statistical analysis was performed by Kolmogorov-Smirnov exact test. (D) The level of D220 DENV-2 RNA in spleen was measured by RT-qPCR using primers for the D220 NS5 region (*n* = 6 per group). Data are shown as the mean ± SEM. Statistical analysis was performed by Kolmogorov-Smirnov exact test. (E) Mean percent body weight change compared with each mouse’s weight on day 0 (*n* = 6 per group). Data are shown as the mean ± SEM. Kolmogorov-Smirnov exact test was used for statistical analysis. Significance was not reached on any day. L.D., limit of detection; ns, not significant.

DENV infection leads to the activation of innate immune responses involving several pattern recognition receptors (PRRs) including Toll-like receptor (TLR) 3,^45^ TLR7,^46^ and the RLRs, RIG-I and MDA5,^47^ among others. IRF3 and IRF7, the two family members with the greatest structural homology, are principal mediators of type I IFN induction, reviewed in.^48^ We infected *Irf3/7*^*−/−*^ mice with D220 and then treated them with LNP-DI290, empty LNP or buffer (Figure 6A). Viremia in serum at more than 2.5 log_10_ CCID_50_/mL and viral titer in spleen tissue at more than 3 log_10_ CCID_50_/mL was detected after 3 d.p.i. (Figures 6B and 6C). However, in LNP-DI290-treated mice, no virus was detected in serum or spleen samples. We measured viral RNA in total spleen RNA samples from all mice, but no viral RNA was detected in mice treated with LNP-DI290 (Figure 6D), an outcome that suggested inhibition of virus replication by a robust IFN response. As observed for *Ifnar*^*−/−*^ mice, *Irf3/7*^*−/−*^ mice infected with DENV alone and mice treated with LNP-290 did not show significant differences in weight (Figure 6E), indicating that DI290 treatment did not exacerbate DENV-induced pathogenesis or induce unexpected adverse event during the course of the experiment. Given the importance of RIG-I and MDA5 in establishing innate immune responses to DENV infection,^47^ this outcome was consistent with a previous study that showed that IRF3 and IRF7 are not solely responsible for protection of mice from DENV infection, as IRF1 can mediate IFN responses in their absence.^49^

### Activation of antiviral responses after treatment of DENV DIPs and LNP-DI290 *in vivo* and *in vitro*

To investigate host responses after treatment with DENV DIPs/LNP-DI290, we used RNA sequencing (RNA-seq) to quantitatively measure the expression levels of transcripts in spleen tissues from both C57BL/6J and *Ifnar*^*−/−*^ mice treated with LNP-DI290 or empty LNPs. In addition, we analyzed RNA from LNP-DI290 and empty LNP-treated MDMs. We also included DENV DIP-treated MDMs to compare induction patterns after LNP treatment versus DIP treatment.

**Figure 7:**
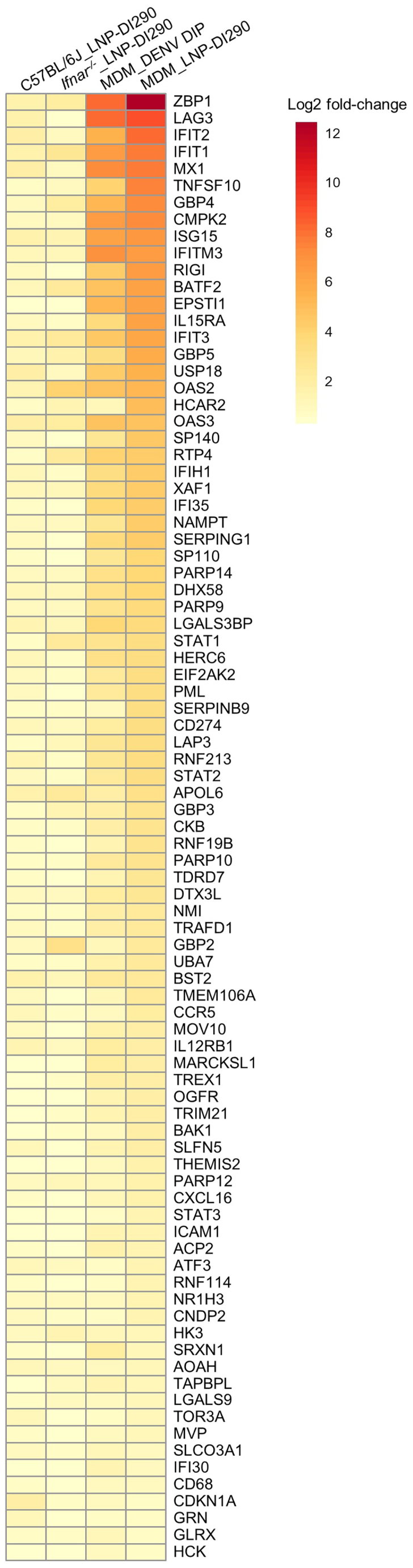
Results of EdgeR analysis Heatmap shows log_2_ fold-changes of 87 DEGs that were significantly upregulated in all four experimental groups. DEGs are ordered by log_2_ fold-change in the LNP-DI290 treated MDM (MDM_LNP-DI290) group.

Approximately 10% of genes in the human genome have the potential to be regulated by IFNs.^50^ The RNA-seq analysis revealed substantial transcriptional alterations in host cells induced by DENV DIP/LNP-DI290 treatment (Table S1. RNA-seq results of DEGs in Ifnar−/− mice treated with either empty LNP or LNP-DI290, Table S2. RNA-seq results of DEGs in human MDMs treated with either empty LNP or LNP-DI290, Table S3. RNA-seq results of DEGs in human MDMs treated with either buffer or DENV DIP, Table S4. RNA-seq results of DEGs in C57BL/6J mice treated with either empty LNP or LNP-DI290). A total of 87 upregulated genes were identified in both mouse spleen tissues and human MDMs (Figure 7). Notably, more than 45 shared upregulated genes in response to these treatments represented ISGs, including RIG-I, MDA5 (also called IFIH1), guanylate binding protein 2 (GBP2), Z-DNA-binding protein 1, myxoma resistance 1 (MX1), ISG15, IFN-induced protein with tetratricopeptide repeats (IFIT) proteins 1–3 (IFIT1-3), IFN-induced transmembrane protein 3 (IFITM3), and 2′5′-oligoadenylate synthetase 2 and 3 (OAS2-3), among others. ISGs upregulated in MDMs and mice in this study, which were previously reported to inhibit DENV replication, including GBP1/2,^51^^,^^52^ ISG15,^53^ OAS3,^54^ IFITM3,^55^ BST2 (teherin),^55^^,^^56^ and CMPK2.^57^ In addition, PRRs, including RIG-I, MDA5 (IFIH1), and DExH-box helicase 58 (DHX 58, also known as LGP2), were upregulated by DI290 RNA in *Ifnar*^*−/−*^ mice where IFN-α/β signaling is impeded, suggesting a role for IFN-λ.^58^ Of note, IFN-γ is more prominent in activating GBPs when compared with IFN-α/β.^59^ Notably, although the antiviral ISG *Mx1* was an upregulated DEG, the *Mx1* allele in these mouse strains is non-functional.^60^

**Figure 8:**
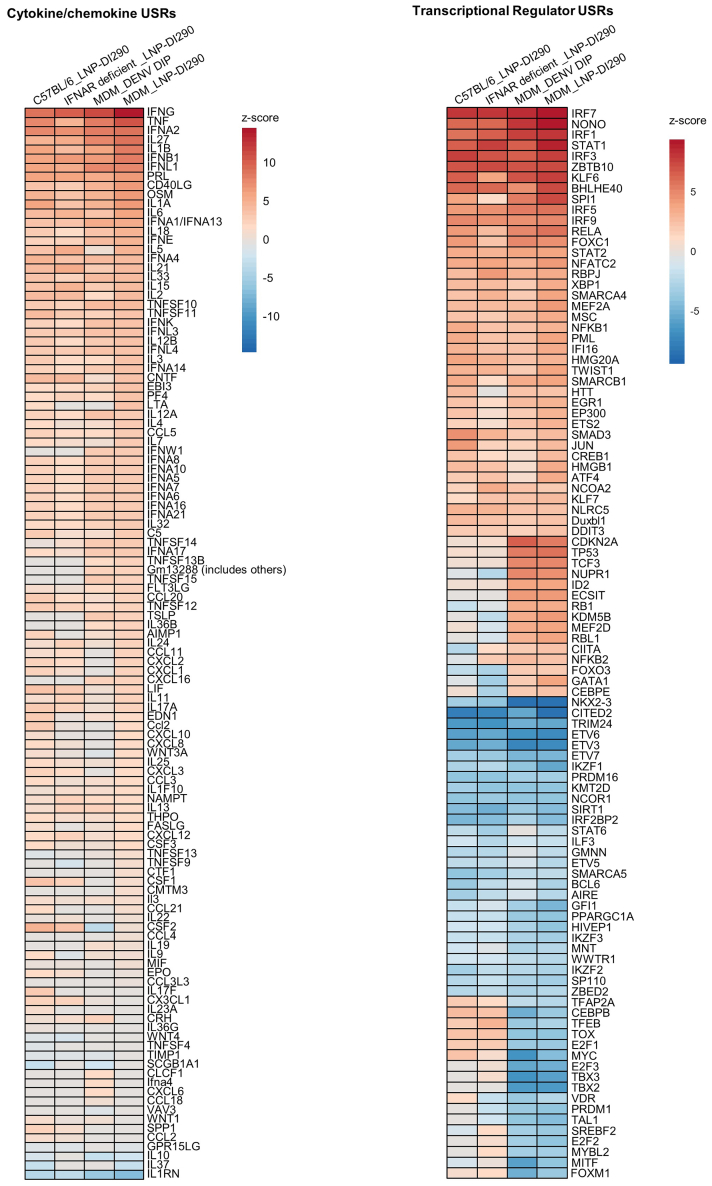
Results of IPA core analysis (A) Heatmap shows activation z-scores of 117 cytokine/chemokine USRs that displayed significant activation or inhibition in at least one experimental group. Any USR that was not significant in a particular group was given a *Z* score of zero. USRs are ordered by *Z* score in the MDM group treated with LNP290 (MDM_LNP-DI290). (B) Heatmap shows activation z-scores of transcription factor USRs that displayed significant activation or inhibition in at least one experimental group. The 100 most activated/inhibited USRs across all groups are shown. Any USR that was not significant in a particular group was given a *z-*score of zero. USRs are ordered by *z-*score in the MDM_LNP-DI290 group.

To further explore the cellular responses, the outcomes of RNA-seq analysis were subjected to ingenuity pathway analysis (IPA) to identify upregulated or downregulated upstream regulators (USRs) common to both DIP or LNP-290 treatments. Heatmaps show activation z-scores of cytokine/chemokine USRs (Figure 8A) and transcriptional regulators USRs (Figure 8B) that were significantly upregulated. The analysis identified activation of 117 cytokines/chemokines that include IFN-γ, tumor necrosis factor (TNF), IFN-α (IFNA1 and IFNA2), IFN-β (IFNB1), and IFN-λ (IFNL1). Interleukin (IL)-27 was an upregulated differential expressed gene (DEG) in *Ifnar*^*−/−*^ mice treated with LNP-290 (Table S1). The activation *Z* score of IL-27 is consistent with the regulation of both innate and adaptive immunity.^61^ IL-27 can induce IFN-γ and inflammatory mediators from T lymphocytes and innate immune cells.^62^ The high activation *Z* score of IRF USRs included IRF1, IRF3, IRF5, IRF7, and IRF9 (Figure 8B). *Irf1* was also an upregulated DEG in *Ifnar*^*−/−*^ mice, while *Irf1*, *Irf2*, and *Irf7* were upregulated DEGs in MDMs treated with LNP-290 (Table S2). Other activation *z*-scores for transcription factors include Zbtb10, which is involved in dendritic cell activation of STAT1, STAT2, and NF-κB (NFKB1, NFKB2), which regulate inflammatory responses.^63^ *STAT1* was an upregulated DEGs in MDMs and *Ifnar*^*−/−*^ mice (Table S1. RNA-seq results of DEGs in Ifnar−/− mice treated with either empty LNP or LNP-DI290, Table S2. RNA-seq results of DEGs in human MDMs treated with either empty LNP or LNP-DI290, Table S3. RNA-seq results of DEGs in human MDMs treated with either buffer or DENV DIP). Taken together, the observed modulation of key inflammatory regulators, particularly the activation of IFNs and associated immune effectors, demonstrates that DENV DIP/LNP-DI290 treatment triggers a robust innate immune response when stimulated by DI290 RNA *in vitro* and *in vivo*.

## Discussion

While vaccines like Dengvaxia (Sanofi) and QDENGA (Takeda) represent significant progress in dengue prevention, treatments are desperately needed with climate change and unplanned urbanization predicted to further increase the global burden.^64^ Although there are promising candidates for antiviral development, no specific drugs have been approved, largely due to issues with efficacy and toxicity. New inhibitors of DENV, such as JNJ-A07, are in development.^65^ Nonetheless, ongoing research into combination therapies, host-targeted strategies, and innovative approaches like DI RNAs holds promise for improved dengue treatment in the future.^65^^,^^66^ Herein, we show that DI290, a naturally derived DENV-2 DI RNA,^4^ triggers host cell antiviral responses and effectively suppresses DENV-2 infection both *in vitro* and *in vivo*. Previously, we demonstrated that DI290 effectively inhibited all DENV serotypes *in vitro*.^5^ DI290 inhibited DENV-2 replication in human cell types targeted by DENV and in two mouse models. However, whether DI290 can inhibit all DENV serotypes *in vivo* remains to be established. DI RNAs derived from other RNA viruses are known to initiate IFN responses, inhibiting viral replication and promoting an antiviral state in infected cells.^15^^,^^21^^,^^23^^,^^27^^,^^30^^,^^67^^,^^68^^,^^69^ In this study, DI290 RNA delivered via DIPs or LNPs also activated innate immune responses that are reported to inhibit DENV replication.^51^^,^^52^^,^^53^^,^^54^^,^^55^^,^^56^^,^^57^ Future studies will evaluate whether DI290 can inhibit heterologous RNA viruses *in vivo*, such as Zika virus and IAV, similar to the broad-spectrum antiviral activity demonstrated by eTIPs using a DVG derived from poliovirus.^23^

Interestingly, analysis of mRNA from treated and untreated cells *in vitro* showed that DIPs significantly upregulated IFN-β and RIG-I levels in MDMs after 2 h (Figure 1). In contrast, MDMs treated with LNP-290 showed upregulated levels of IFNs and ISGs only after 24 h (Figure 3). These results suggest that delivery vehicles, whether DIPs or LNPs, can influenced the kinetics of IFN activation, with a delayed, or less efficient response observed with LNP-DI290. DIPs are a DENV virus-like particles (VLPs) endowed with viral envelope protein, enabling endosomal-mediated entry into the cell and endosomal escape. While LNPs are internalized into cells via both clathrin-dependent and independent endocytosis,^70^^,^^71^ with the process of endosomal escape inefficient and considered a bottleneck.^72^ The difference in innate immune activation kinetics was not dose related as MDMs treated with LNP-290 received more RNA compared with DIPs (25 pg vs. 0.2 fg per cell). Overall, the *in vitro* results suggest that compared with LNPs, DIPs are a more efficient DI290 delivery vehicle for induction of innate immunity. Clearly, LNP and LNP technologies are evolving rapidly so future improvements in LNP technology may overcome these bottlenecks. Such improvements may include strategizes for enhanced endosomal escape,^73^ and alternative LNP design.^74^

*Ifnar*^*−/−*^ mice are susceptible to DENV infection due to impaired IFN-α/β signaling and diminished antiviral responses.^43^^,^^75^ Our study indicates that DI290 upregulated IFN responses including IFN-λ and IFN-γ signaling in all systems analyzed. IFN-λ, a type III IFN, activates antiviral gene expression programs similar to IFN-α/β (type I IFNs) and is expressed by MDM cells (Figures 1 and 3), which are a cell type targeted by DENV. IFN-γ, a type II IFN, is largely expressed by T cells and natural killer cells and can be upregulated by IL-15 that is typically secreted by monocytes and macrophage.^76^ RNA-seq analysis showed IL-15 mRNA was a strongly upregulated DEG in MDMs treated with LNP-DI290 and DENV DIPs (Tables S2 and S3). Also, GBPs are the ISGs most strongly upregulated by IFN-γ ISGs compared with IFN-α/β,^59^ which were strongly upregulated DEGs in all systems (Table S1. RNA-seq results of DEGs in Ifnar−/− mice treated with either empty LNP or LNP-DI290, Table S2. RNA-seq results of DEGs in human MDMs treated with either empty LNP or LNP-DI290, Table S3. RNA-seq results of DEGs in human MDMs treated with either buffer or DENV DIP, Table S4. RNA-seq results of DEGs in C57BL/6J mice treated with either empty LNP or LNP-DI290) including *Ifnar*^*−/−*^ mice where IFN-α/β is absent. IFN-γ′s activation of macrophages and T cells could significantly contribute to limiting DENV dissemination. IFN-γ strongly induces the expression of ISGs in macrophages, such as RIG-I, MDA5, and ISG15, which are important in recognizing viral RNA and inhibiting viral replication. IFN-γ can boosts the phagocytic capacity of macrophages, allowing them to engulf and clear infected cells, including those infected by DENV.^77^ By clearing infected cells, IFN-γ-activated macrophages can reduce the reservoir of virus available for replication and subsequent spread. IFN-γ enhances the capacity of macrophages to present viral antigens via major histocompatibility complex class I and II molecules.^78^^,^^79^ This activation improves macrophage interactions with T cells, this could lead to a more robust adaptive immune response against DENV. Notably, IFN-γ signaling has been shown to be protective against DENV pathogenesis in vaccinated individuals,^80^ and in *Ifnar*^*−/−*^ mice.^75^ Future research plans include testing DI290 in AG129 mice, which lack both type I and type II interferon (IFN-γ) responses. This will help to elucidate the role of IFN-γ in DI290-mediated inhibition of DENV infection.

Overall, IFN-driven antiviral responses observed in *Ifnar*^*−/−*^ mice and MDM cells are consistent with greatly reduced or undetectable viremia in infected *Ifnar*^*−/−*^ mice and *Irf3/7*^*−/−*^ mice treated with LNP-DI290. Our report supports a previously study of IRF3/5/7 triple knockout mice infected with DENV, which controlled DENV infection due to IFN-γ signaling via IRF1 to stimulate antiviral responses.^49^ While we did not undertake RNA-seq analysis of *Irf3/7*^−/−^ mice samples, both MDM cells and *Ifnar*^*−/−*^ mice treated with LNP-290 upregulated IRF1 (Tables S1 and S2). The transcription factor IRF1 contributes to both IFN-α/β and IFN-γ signaling to regulate viral infection.^81^^,^^82^^,^^83^^,^^84^^,^^85^^,^^86^ Therefore, the evidence here supports the notion that DI290 RNA exerted its effect in *Ifnar*^*−/−*^ mice by broadly upregulating IFN mediated antiviral activity.

The evidence in this study did not support that DI290 inhibited DENV through a viral interference mechanism. DI RNAs belong to the class of defective viral genomes, which emerge during viral replication and are packaged and released as non-infectious VLPs alongside infectious wild-type viruses. The presence of these VLPs in a virus stock is associated with reduced viral replication and virulence, indicating attenuation.^7^^,^^10^^,^^27^^,^^87^^,^^88^^,^^89^^,^^90^ Previously, DI290 was shown to be replication competent and was packaged into DIPs *de novo*.^5^ Although the DENV stocks used for infections contained undetectable levels of DI290 (Figure S7), the generation of DI290 in DENV-infected mice was observed (Figure 5F). However, similar levels of DI290 were measured across all LNP-290-treated mouse groups, including uninfected mice (Figure 5F). A role of DI290 in viral interference may be complicated by the many upregulated ISGs that oppose viral RNA synthesis, such as ISG15,^53^ IFIT1,^91^^,^^92^ and OAS enzymes.^54^ These antiviral activities are consistent with the reduced viral genomic RNA levels and inhibited viral replication observed in infected *Ifnar*^*−/−*^ mice treated with LNP-290, but they may have also impacted DI290 levels *in vivo*. Further research will be required to understand the interplay between antiviral responses and its effect on DI290 replication dynamics especially *in vitro* where individual ISGs could be knocked down to see if this will reduce or enhance DI290 replication.

Activation of IFN-mediated signaling pathways by DI RNA may lead to unintended consequences that could be harmful. Excessive activation of the immune system, particularly if sustained, can result in immunotoxicity such as cytokine storms. These are typically characterized by the massive release of several pro-inflammatory cytokines, including Il-2, IL-6, and TNF. While IPA analysis identified these cytokines as USR (Figure 8), the corresponding mRNAs were not significantly upregulated DEGs in any of the systems tested (Table S1. RNA-seq results of DEGs in Ifnar−/− mice treated with either empty LNP or LNP-DI290, Table S2. RNA-seq results of DEGs in human MDMs treated with either empty LNP or LNP-DI290, Table S3. RNA-seq results of DEGs in human MDMs treated with either buffer or DENV DIP, Table S4. RNA-seq results of DEGs in C57BL/6J mice treated with either empty LNP or LNP-DI290). Overall, LNP-DI290-treated mice did not experience significant weight loss compared with untreated infected mice, indicating that DI290 treatment did not exacerbate DENV-induced pathogenesis or promote unexpected adverse events.

In summary, we have demonstrated that DI290 RNA delivered by DIPs or LNP-290 activates innate immune responses in primary human macrophages and fibroblasts, primary targets for DENV infection, and strongly inhibits DENV infection. LNP-DI290 strongly inhibits DENV replication in *Ifnar*^*−/−*^ mice and *Irf3/7*^−/−^ mice. Control of DENV replication was associated with solid upregulation of antiviral ISGs. Future studies to investigate if prophylactic LNP-DI290 application is protective, if administration at a later time point after infection provides protection from infection, or to benchmark DI290 antiviral activity compared with other RIG-I agonists is warranted but a different approach and animal model may be necessary. These findings position DI290 as a promising therapeutic strategy for DENV, capable of robustly stimulating immune defenses even in the absence of key IFN responses.

## Materials and methods

### Ethics statement

All mouse work was conducted in accordance with the “Australian code for the care and use of animals for scientific purposes” as defined by the National Health and Medical Research Council of Australia. All animal procedures were conducted in a dedicated suite in a biosafety level-3 (PC3) facility at the QIMR Berghofer Medical Research Institute (Australian Department of Agriculture, Water and the Environment certification Q2326 and Office of the Gene Technology Regulator certification 3445) as previously described.^93^ All work was approved by the QIMR Berghofer Medical Research Institute Animal Ethics Committee (P2277, A2002-600). Mice were euthanized using carbon dioxide asphyxiation. Overt clinical signs of mice were scored, as described in,^94^ on a scale of 0–3 (diseases scores) according to posture, activity and fur ruffling, with a score of 0 meaning no clinical signs were observed.

Breeding and use of GM mice were approved under a Notifiable Low Risk Dealing (NLRD) Identifier: NLRD_Harrich_Aug_2023: NLRD 1.1(a), NLRD 2.1(d), NLRD 2.1(j), NLRD 2.1(l).

### Cell culture

Vero E6 (ATCC CRL-1586) and HFF-1 (ATCC SCRC-1401) cells were maintained in DMEM (Thermo Fisher) supplemented with 10% (v/v) fetal bovine serum (FBS) (Thermo Fisher) and 1% (v/v) penicillin-streptomycin (Thermo Fisher). DENV DIP-producing cell line, HEK-DI-290-ORF, was cultured in HEK GM Serum-Free Medium (Sartorius) supplemented with 1% (v/v) penicillin-streptomycin, 1% (v/v) GlutaMAX-I (Thermo Fisher), and 50 nM PEP005 (Adooq Bioscience) during DENV DIP production.^6^ The human monocytic cell line THP-1 was maintained in RPMI 1640 medium supplemented with 10% (v/v) FBS and 1% (v/v) penicillin-streptomycin. THP-1 cells were differentiated into macrophage-like cells by stimulation of 50 ng/mL phorbol myristate acetate (PMA) for 48 h. The THP-1 cells were then cultured for 24 h with PMA-containing medium replaced with a complete medium. All cells were incubated at 37°C in a humidified 5% CO_2_ atmosphere. C6/36 cells were grown in DMEM supplemented with 10% (v/v) FBS and 1% (v/v) penicillin-streptomycin. Cells were checked for mycoplasma using MycoAlert Mycoplasma Detection Kit (Lonza Bioscience). Cell lines are routinely authenticated in-house by short tandem repeat profiling.

### Isolation of monocytes from peripheral blood mononuclear cells

Peripheral blood mononuclear cells (PBMCs) were isolated from a healthy donor’s buffy coat supplied by the Australian Red Cross Blood Service using Ficoll density gradient centrifugation as previously described.^95^ After PBMC isolation, cells were washed and resuspended in PBS containing 2% FBS and 1 mM EDTA. Monocytes were isolated from PBMCs using EasySep Human CD14 Positive Selection Kit according to the manufacturer’s instructions (Stemcell Technologies).

### Differentiation of human monocyte to MDMs

MDMs were differentiated as previously described.^96^ Briefly, isolated monocytes were cultured in RPMI 1640 (Thermo Fisher) supplemented with 10% (v/v) FBS, 1% (v/v) penicillin-streptomycin, 10 ng/mL macrophage colony stimulating factor (M-CSF) (Thermo Fisher) and 1 ng/mL granulocyte-M-CSF (GM-CSF) (Thermo Fisher) for 5 days. Medium was changed every 2 days.

### DENV DIP production, precipitation, and chromatographic purification

HEK-DI-290-ORF cells were seeded at 2.5 × 10^5^ cells/mL and grown in a ProCulture glass spinner flask (Corning) using a magnetic stir plate (Thermo Fisher) set at 65 rpm. After 48 h, the culture supernatant containing DENV DIPs was collected, filtered through a 0.22-μm filter (Merck Millipore), and the clarified supernatant was stored at 4°C. The clarified supernatant was processed through the tangential flow filtration (TFF) system (AKTA Flux S, Cytiva), which was equipped with a 100 kDa molecular weight cut-off filter (UFP-100-C-H24LA hollow fiber cartridge) and equilibrated with sterile 1× PBS. Upon equilibration, the clarified supernatant was added to the TFF chamber and flowed through 54 mL/min and a permeate pump rate of 5 mL/min. The transmembrane pressure was maintained below 0.5 bar. The resulting concentrated retentate was then filtered through a 0.22-μm filter and added with stabilizing buffer containing 2% (v/v) gelatin hydrolysate (Merck G0262) and 5% (v/v) sorbitol (Merck S1876) before CHT ceramic hydroxyapatite (Bio-Rad Laboratories) chromatographic purification of DENV DIPs.

CHT chromatography column purification was performed as previously described.^6^ The resulting purified DENV DIPs were then concentrated using an Amicon Ultra-15 centrifugal filter unit (50 kDa cut-off) until the volume was reduced to approximately 1–1.5 mL. Quantification of DI290 RNA copies was performed by qPCR analysis.

### *In vitro* synthesis of DI290 RNA

The pPUC57-CMV-DI290-HDVr plasmid, synthesized by GenScript, was used as the template for generating the DI290 PCR product. The PCR product was then used as a template to generate DI290 RNA *in vitro* using T7 RiboMAX Large Scale RNA Production System (Promega) according to the manufacturer’s instructions. The primer sequences to generate the DI290 PCR product are available on request.

### LNP-DI290 formulation

LNPs encapsulating DI290 RNA, were created using the hydration method.^97^ Briefly, required amounts of lipid and PEG2000-C16Ceramide were mixed with DI290 RNA at an N:P ratio of 4 in a sucrose-containing water/tert-butanol (1:1 v/v) co-solvent system. DOTAP, cholesterol, DOPE, and PEG2000-C16Ceramide with a molar ratio of 50:35:5:10 was used. The mixture was then snap-frozen and freeze-dried overnight. Freeze-dried matrix was then hydrated with sterile water immediately before use at a concentration of 0.1 mg/mL D1290 final.

### Cell viability assessment

The Cell Titer 96 Aqueous One solution cell proliferation assay (MTS assay, Promega) was used to quantify relative cell viability. Assays were performed according to the manufacturer’s instructions. Briefly, 1,000 cells per well were seeded into a 96-well plate. Cells were treated with varying concentrations of Rux for up to 72 h. MTS reagent (20 μL) was added into each well and incubated at 37°C with 5% CO_2_ for 1 h. Absorbance was measured at 490 nm.

### Analysis of DENV DIP and LNP-DI290 antiviral activity *in vitro*

HFF-1, MDM, and THP-1 cells were seeded in a 48-well plates with 10,000 cells per well. THP-1 cells were induced for differentiation, as described above. The next day, the cells were infected with DENV-2 (MOI = 0.1 CCID_50_ per cell for THP-1 and MOI = 1 CCID_50_ per cell for THP-1 and MDM). After 3 h, the cells were washed with 1× PBS and incubated with either culture medium alone, medium containing DENV DIP (at a dosage equivalent to 1,000 DI290 RNA copies [=0.2 fg] per cell), empty LNP or LNP-DI290 (at a dosage equivalent to 1.6 × 10^8^ DI290 RNA copies [=25 pg] per cell). At 3 days post-infection, the viral titers were determined by CCID_50_ assays as previously described.^6^^,^^98^

For cells receiving the JAK inhibitor Rux (Merck), HFF-1 cells were pre-incubated with Rux (5 μM) for 1 h and then treated with LNP-DI290 (equivalent to 25 pg DI290 RNA per cell) or empty LNP in the presence or absence of Rux (5 μM) for 24 h. Total RNA was extracted from the cells and the levels of ISG15 mRNA were quantified by RT-qPCR. The fold change relative to the untreated control was calculated. For infection studies, HFF-1 cells were pre-treated with Rux (5 μM) for 1 h and then infected with DENV-2 (MOI = 0.1 CCID_50_/cell) in the presence or absence of Rux. After 3 h, the cells were washed with 1× PBS and incubated with culture medium containing LNP-DI290 (equivalent to 25 pg DI290 RNA per cell) in the presence or absence of Rux. DENV-2 titers in culture supernatant were measured by CCID_50_ assay at 3 days post-infection. In a separate experiments, HFF-1 cells were treated with LNP-DI290 (equivalent to 25 pg DI290 RNA per cell) or empty LNP in the presence or absence of anifrolumab (10 μg/mL) or IgG1 control antibody (10 μg/mL) for 24 h. Total RNA was extracted from the cells and the levels of ISG15 mRNA were quantified by RT-qPCR. For infection experiments, HFF-1 cells were infected with DENV-2 (MOI = 0.1 CCID_50_/cell) in the presence or absence of anifrolumab. After 3 h, the cells were washed with 1X PBS and incubated with culture medium containing LNP-DI290 (equivalent to 25 pg DI290 RNA per cell) in the presence or absence of anifrolumab. DENV-2 titers in culture supernatant were measured by CCID50 assay at 3 days post-infection.

### Gene expression analysis

HFF-1, MDM, and THP-1 cells were seeded at a density of 50,000 per well in a 48-well plate. THP-1 cells were induced for differentiation, as described above. The next day, the cells were treated with culture medium only or medium containing either DENV DIP (equivalent to 1,000 DI290 RNA copies [=0.2 fg)/cell], empty LNP or LNP-DI290 (equivalent to 1.6 × 10^8^ DI290 RNA copies [=25 pg]/cell). Total RNA from cells was extracted after 2, 24, 48, and 72 h of treatment using RNeasy Kit (Qiagen) and mRNA expression levels of Ribosomal Protein L13a (RPL13A), IFN-β, RIG-I, and ISG15 were determined using Luna Universal One-Step RT-qPCR Kit according to the manufacturer’s instructions (New England Biolabs). The primer sequences are available upon request. The data was normalized to RPL13A as the reference gene^99^ and presented as fold change relative to untreated control cells using ΔΔC_t_ method.

### ELISA

HFF-1 cells were seeded at a density of 20,000 per well in a 48-well plate. The next day, the cells were treated with culture medium only or medium containing either DENV-2 (MOI = 0.1 CCID_50_ per cell), DENV DIP (equivalent to 1,000 DI290 RNA copies [=0.2 fg]/cell), or a mixture of DENV-2 and DENV DIP. The culture supernatants and cells lysates were collected at 2, 24, 48, and 72 h after treatment. The levels of IFN-β in the culture supernatants and ISG15 in the cell lysates were measured using human IFN beta simplestep ELISA and human ISG15 ELISA kits (Abcam), according to the manufacturer’s instructions.

### Mouse infection

All mice were bred in-house and housed at QIMR Berghofer Medical Research Institute, Brisbane, QLD, Australia. *Ifnar*^*−/−*^ mice,^43^ and *Irf3/7*^*−/−*^ mice,^100^ were infected i.p. with 1 × 10^4^ CCID_50_ of DENV-2 strain D220,^101^ for 4 h. Mock-infected mice were injected i.p. with PBS. After 4 h infection, the mice were treated i.p. with either LNP-DI290 containing 15 μg of DI290 RNA or empty LNPs. After treatment, body weight and general conditions were monitored daily for up to 3 days. The mice were euthanized using CO_2_ at 3 days after infection. Blood was harvested through heart puncture and the spleen was collected and homogenized in 1 mL RPMI 1640 containing 2% FBS. The homogenate (200 μL) was mixed with Trizol (Thermo Fisher) for RNA extraction, followed by quantification of viral RNA using RT-qPCR. Virus titration in both blood and spleen samples was determined by CCID_50_ assays as previously described.^6^^,^^98^

### Quantification of viral RNA from spleen samples

After Trizol extraction, the RNA pellet was resuspended in 50 μL of RNase-free H_2_O and 1 μL of the resuspended samples was used for quantification of the DENV-2 D220 NS5 RNA regions using Luna Universal One-Step RT-qPCR Kit (New England Biolabs) in a 10-μL reaction volume. The thermal cycling conditions were as follows: 15 min at 55°C, 2 min at 95°C and 40 cycles of 5 s at 95°C, 45 s at 55°C (data acquisition), and 15 s at 72°C. The primer sequences are available on request.

### RNA-seq sample library preparation and analysis

For the preparation of MDM mRNA, MDMs were seed in 48-well plates with 30,000 cells per well and treated with either culture medium alone, medium containing DENV DIP (at a dosage equivalent to 0.2 fg DI290 RNA copies per cell, *n* = 3), empty LNP (*n* = 3) or LNP-DI290 (at a dosage equivalent to 25 pg DI290 RNA copies per cell, *n* = 3). After 24 treatments, total RNA was extracted from the cells using RNeasy Kit (Qiagen) according to the manufacturer’s instructions. For mRNA preparation from mouse spleens, C57BL/6J (*n* = 6) and *Ifnar*^*−/−*^ (*n* = 5) mice were i.p. treated with empty LNP or LNP-DI290 (at a dosage equivalent to 15 μg DI290 RNA per mouse). After 24 h of treatment, whole spleens were harvested, preserved in RNA*later* solution (Thermo Fisher) and homogenized in Trizol (Thermo Fisher). Total spleen RNA was then extracted following the manufacturer’s instructions.

RNA concentration and quality were measured using TapeStation D1kTapeScreen assay (Agilent). cDNA libraries were generated using Illumina TruSeq Stranded mRNA library prep kit and were sequenced using an Illumina Nextseq 2000 Sequencing System to produce 75nt paired-end reads. Sequence reads were aligned to either the GRCm39 v33 mouse or the GRCh38 v44 human reference genomes obtained from Gencode, using STAR aligner v2.7.10. Aligned read counts were calculated for each annotated gene using RSEM v1.3.1, and differential expression was estimated with EdgeR v3.42.4 using the statistical software, R v4.2.0. Only genes with at least 0.5 counts per million (approximately 10 aligned reads) were included in the test for differential expression. Significantly DEGs were analyzed using IPA v107193442 (QIAGEN). IPA accepts DEG sets of between 200 and 3,000 genes. Therefore, false discovery rate cut-offs were adjusted to keep DEG sets within this range and roughly comparable between experimental groups. q < 1 × 10^−2^ was used for the C57BL/6J group; q < 1 × 10^−5^ was used for the IFNAR-deficient group; q < 1 × 10^−3^ was used for the DIP-treated MDM group; and q < 1 × 10^−5^ was used for the LNP-DI290-treated MDM group.

### Statistical analysis

Statistical analysis was performed using IMB SPSS Statistics (IMB Corp.) and GraphPad Prism (GraphPad software). When the difference in variance was >4, skewness was <−2 or kurtosis was >2, the data were considered non-parametric and the Kolmogorov-Smirnov exact test was performed. Otherwise, the one-way ANOVA or student’s t test were used. A *p* value of less than 0.05 was considered significant.

## Data and code availability

All data generated or analyzed during this study are included in this published article and its Supplementary tables and information. Any remaining information can be obtained from the corresponding author upon reasonable request.

## Acknowledgments

This research was generously supported through funding from the 10.13039/100010269Wellcome Trust Innovator Award 219588Z/19/Z to D.H. and the DARPA INTERfering and Co-Evolving Prevention and Therapy (10.13039/100018262Intercept) program to D.H. and J.A. We sincerely thank John Aaskov for the introduction to DENV defective interfering particles field. We also thank Dr. I Anraku for his assistance in managing the PC3 (BSL3) facility at QIMR Berghofer Medical Research Institute and animal house staff from QIMR Berghofer Medical Research Institute for mouse breeding and agistment.

## Author contributions

M.-H.L. and P.M. conducted the experimental work. L.W.-E. and Y.T. synthesized nanoparticles. C.B. conducted RNA-seq analysis. B.T., D.L., and L.L. assisted M-H.L. and P.M. throughout the project. D.H., A.S., N.A.J.M., and M-H.L. conceived, designed, and directed the study. D.H., M-H.L., and P.M. made the figures. D.H., M-H.L., and P.M. analyzed the outcomes and wrote the manuscript. All authors read and approved the manuscript.

## Declaration of interests

D.H., D.L., and M.L. patent number WO2021108863A1 relates to the production of transmissible virus DIPs, particularly those of DENV as well as methods of their production.

## Supplemental information

Document S1. Figures S1–S8

Table S1. RNA-seq results of DEGs in Ifnar−/− mice treated with either empty LNP or LNP-DI290

Table S2. RNA-seq results of DEGs in human MDMs treated with either empty LNP or LNP-DI290

Table S3. RNA-seq results of DEGs in human MDMs treated with either buffer or DENV DIP

Table S4. RNA-seq results of DEGs in C57BL/6J mice treated with either empty LNP or LNP-DI290

Document S2. Article plus supplemental information
