# Supplementary material for: Harnessing defective interfering particles and lipid nanoparticles for effective delivery of an anti-dengue virus RNA therapy
Source: Mol Ther Nucleic Acids. 2024 Dec 12;36(1):102424. doi: 10.1016/j.omtn.2024.102424 (PMC11733052; doi:10.1016/j.omtn.2024.102424)
Supplement: Document S1. Figures S1–S8 [file mmc1.pdf]

OMTN, Volume *36*

Supplemental information

Harnessing defective interfering particles and lipid nanoparticles for effective delivery of an anti-dengue virus RNA therapy

Min-Hsuan Lin, Pramila Maniam, Dongsheng Li, Bing Tang, Cameron R. Bishop, Andreas Suhrbier, Lucy Wales- Earl, Yaman Tayyar, Nigel A.J. McMillan, Li Li, and David Harrich

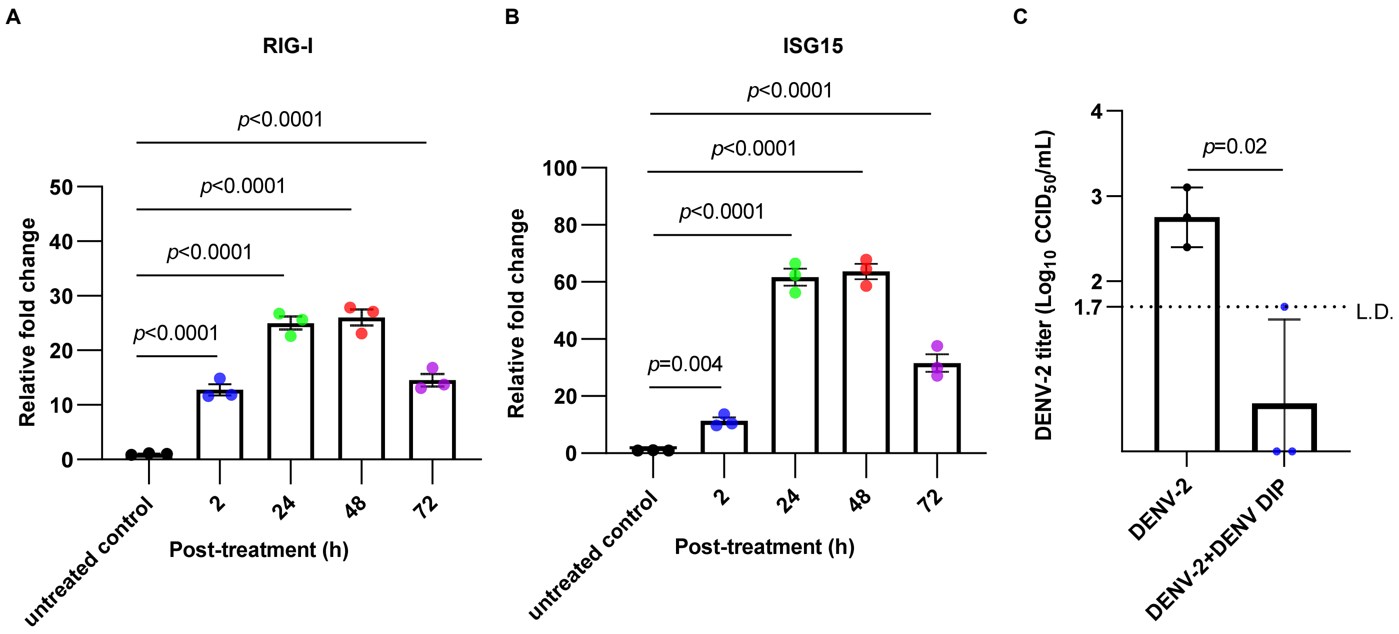

Figure S1. **DIPs upregulate RIG-I and ISG15 in THP-1 cells.** (A-B) THP-1 cells were treated with DENV DIPs (at a dosage equivalent to 1,000 DI290 RNA copies (=0.2 fg) per cell) for 2, 24, 48 and 72 h. Total RNA was extracted from the cells and the levels of RIG-I and ISG15 mRNA were quantified by RT-qPCR. The fold change relative to the untreated control cells was calculated (n=3). (C) THP-1 cells were infected with DENV-2 (MOI=1 $CCID_{50}$ per cell). After 3 h, the cells were washed with 1X PBS and incubated with culture medium containing DENV DIP (at a dosage equivalent to 1,000 DI290 RNA copies (=0.2 fg) per cell). DENV-2 titers in culture supernatant were measured by $CCID_{50}$ assay at 3 days post-infection (n=3). The data are shown as the mean ± SD. P values were calculated using a two-tailed Student’s t test. L.D.: limit of detection.

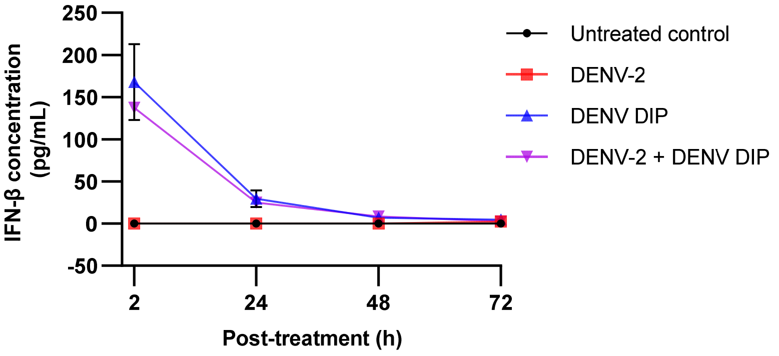

Figure S2. DENV DIPs upregulate IFN-β protein levels in uninfected and DENV-infected cells. HFF-1 cells were treated with DENV-2 (MOI = 0.1 $CCID_{50}$ per cell, red line), DENV DIP (at a dosage equivalent to 1,000 DI290 RNA copies (=0.2 fg) per cell, blue line) or DENV-2 mixed with DENV DIP (purple line). Untreated cells were included as negative control (black line). The concentration of IFN-β protein in culture supernatants was measured at 2, 24, 48 and 72 h post-treatment (n=3). Data are shown as the mean ± SD.

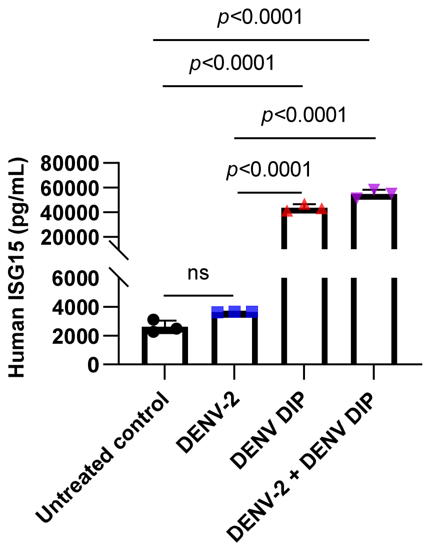

Figure S3. DIPs promote ISG15 levels in uninfected and DENV-infected HFF-1 cells. HFF-1 cells were treated with DENV-2 (MOI = 0.1 $CCID_{50}$ per cell), DENV DIP (at a dosage equivalent to 1,000 DI290 RNA copies (=0.2 fg) per cell) or DENV-2 mixed with DENV DIP. Untreated cells were included as negative control. The concentration of ISG15 protein in cell lysates was measured at 24 h post-treatment (n=3). Data are shown as the mean ± SD. Statistical analysis was performed by one-way ANOVA. ns: not significant.

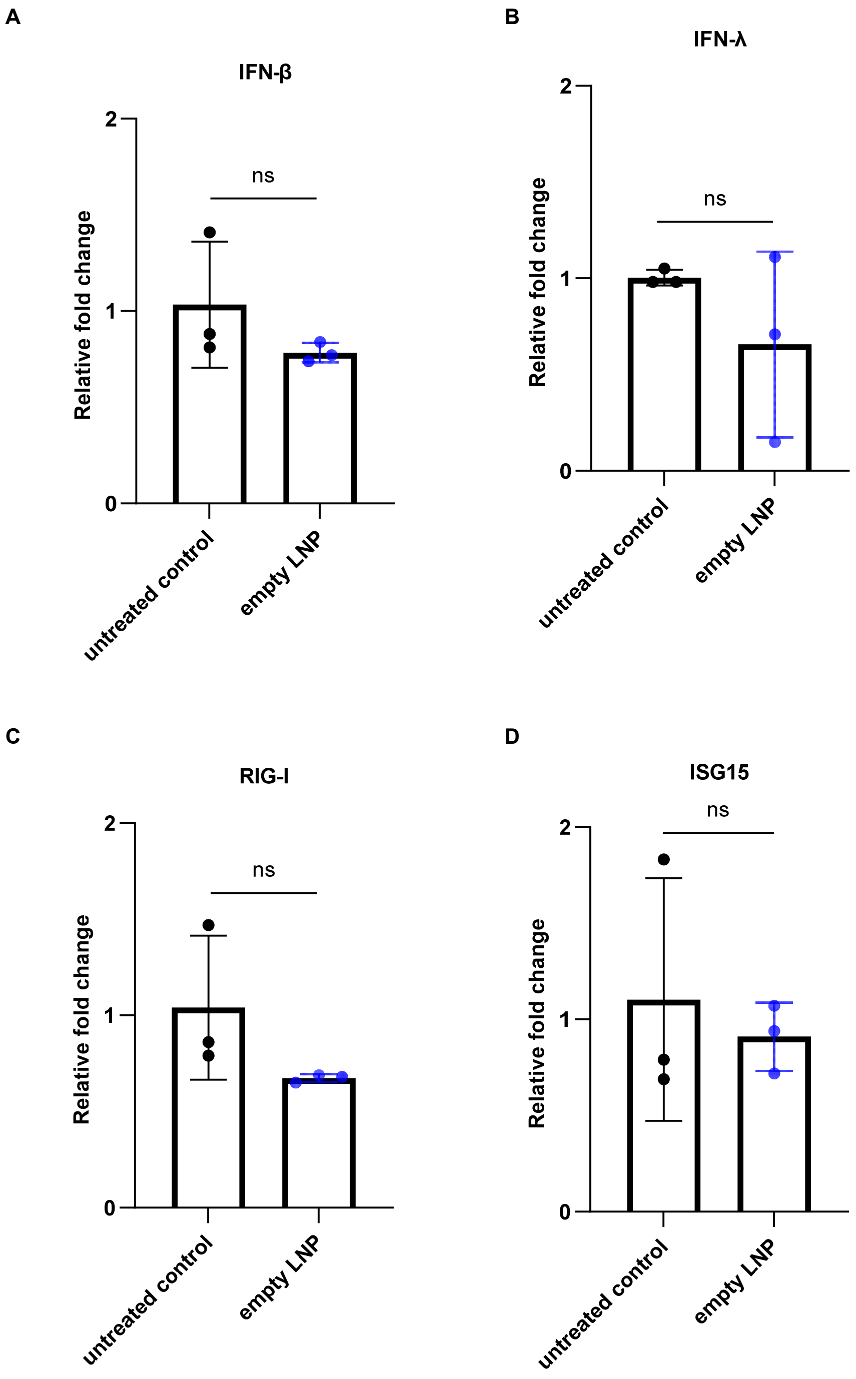

Figure S4. **Empty LNPs do not affect IFN responses in MDM cells.** MDM cells were treated with empty LNP for 24 h. Total RNA was extracted from the cells and the levels of IFN-β, IFN-λ, RIG-I and ISG15 mRNA were quantified by RT-qPCR. The fold change relative to the untreated control cells was calculated (n=3). The data are shown as the mean ± SD. P values were calculated using a two-tailed Student's t test. ns: not significant.

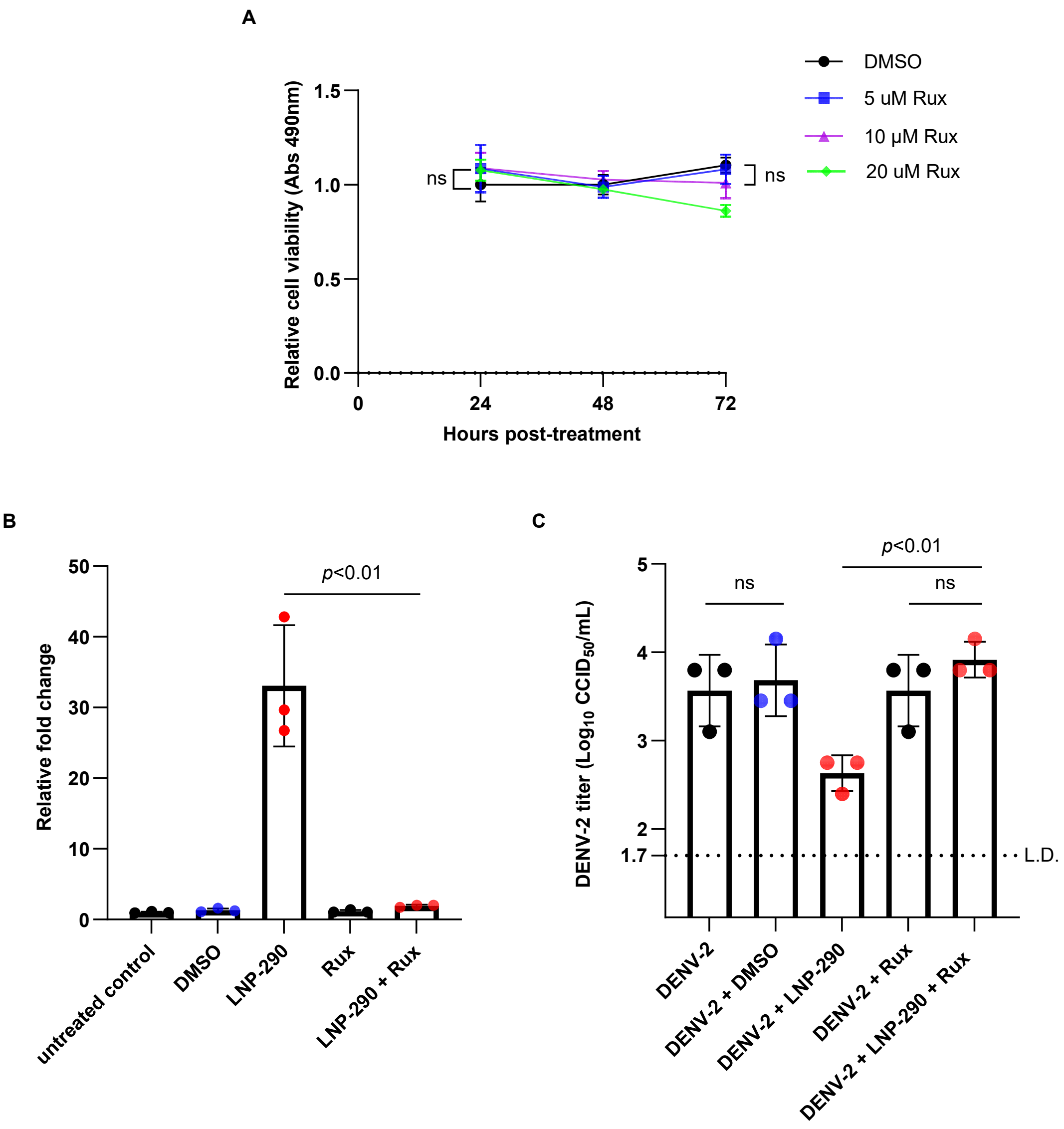

Figure S5. Inhibition of JAK-STAT signalling by Rux counteracts the antiviral effects of LNP-DI290 in HFF-1 cells. (A) HFF-1 cells seeded in 96-well plates were incubated with Rux at concentrations of 5, 10, and 20 μM, or DMSO (vehicle control), for up to 72 hours. Cell viability was assessed using an MTS assay at 24 h intervals, with MTS reagent added and incubated for 1 hour under humidified 5% CO_2 conditions. Absorbance was measured at 490 nm to evaluate cell proliferation. Data are presented as mean ± SD of three replicates. (B) HFF-1 cells were pre-inclubated with Rux (5 μM) for 1 h and then treated with LNP-DI290 (equivalent to 25 pg DI290 RNA per cell) or empty LNP in the presence or absence of Rux (5 μM) for 24 h. Total RNA was extracted from the cells and the levels of ISG15 mRNA were quantified by RT-qPCR. The fold change relative to the untreated control was calculated (n=3). (C) HFF-1 cells were pre-treated with Rux (5 μM) for 1 h and then infected with DENV-2 (MOI=0.1 $CCID_{50}$/cell) in the presence or absence of Rux. After 3 h, the cells were washed with 1X PBS and incubated with culture medium containing LNP-DI290 (equivalent to 25 pg DI290 RNA per cell) in the presence or absence of Rux. DENV-2 titers in culture supernatant were measured by $CCID_{50}$ assay at 3 days post-infection (n=3 per group). Data are shown as mean ± SD. Statistical analysis was performed by one-way ANOVA. ns: not significant. L.D.: limit of detection.

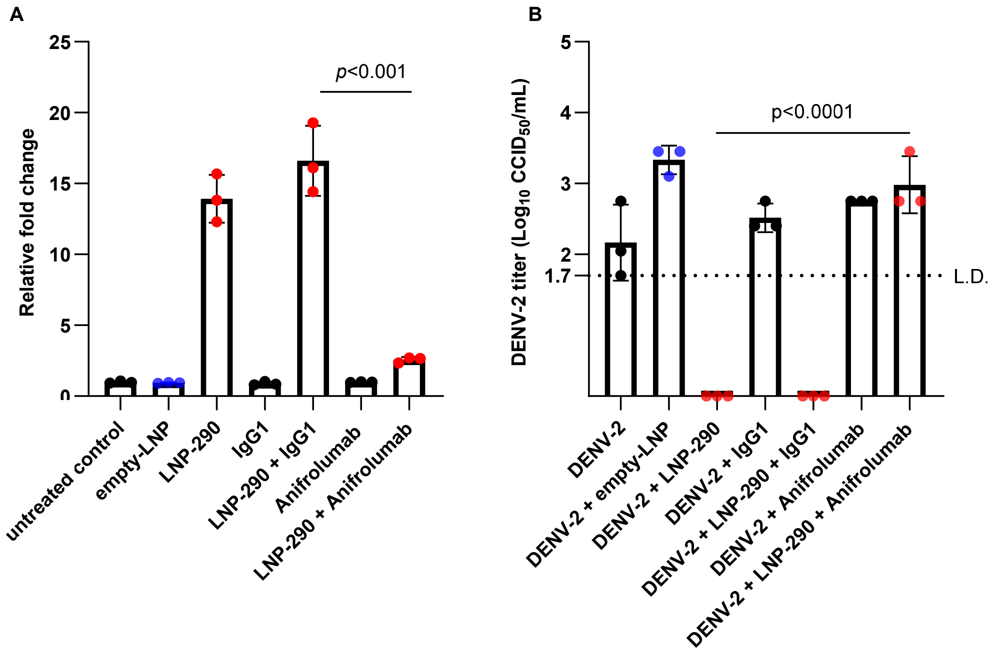

Figure S6. **LNP-DI290's antiviral effects on DENV-2 are mitigated by anifrolumab-mediated inhibition of type I IFN signaling.** (A) HFF-1 cells were treated with LNP-DI290 (equivalent to 25 pg DI290 RNA per cell) or empty LNP in the presence or absence of anifrolumab (10 μg/mL) or IgG1 control antibody for 24 h. Total RNA was extracted from the cells and the levels of ISG15 mRNA were quantified by RT-qPCR. The fold change relative to the untreated control was calculated (n=3). (B) HFF-1 cells were infected with DENV-2 (MOI=0.1 $CCID_{50}$/cell) in the presence or absence of anifrolumab. After 3 h, the cells were washed with 1X PBS and incubated with culture medium containing LNP-DI290 (equivalent to 25 pg DI290 RNA per cell) in the presence or absence of anifrolumab. DENV-2 titers in culture supernatant were measured by $CCID_{50}$ assay at 3 days post-infection (n=3 per group). Data are shown as mean ± SD. Statistical analysis was performed by one-way ANOVA. ns: not significant. L.D.: limit of detection.

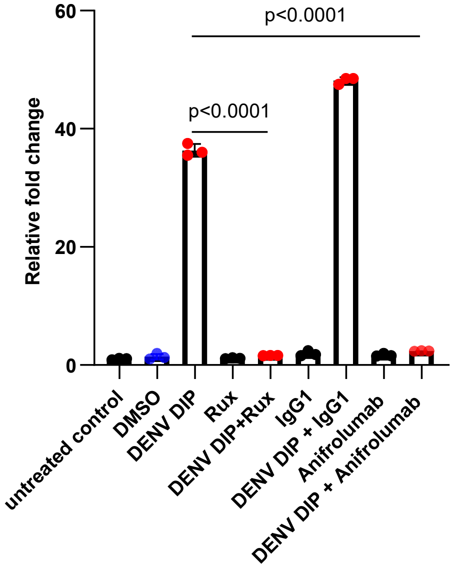

Figure S7. Antiviral effects of DENV DIPs are alleviated by inhibition of JAK-STAT by Rux or inhibition of type I IFN signaling by anifrolumab. HFF-1 cells in experimental groups receiving Rux were pre-incubated with 5 μM of Rux for 1 h and then treated with DENV DIP (equivalent to 1, 000 DI290 RNA copies (=0.2 fg) per cell) in the presence of Rux for 24h. HFF-1 cells in experimental groups receiving anifrolumab or IgG1 control antibody were treated with DENV DIP in the presence or absence of anifrolumab (10 μg/mL) or IgG1 (10 μg/mL) for 24 h. Total RNA was extracted from the cells and the levels of ISG15 mRNA were quantified by RT-qPCR. The fold change relative to the untreated control was calculated (n=3). Data are shown as mean ± SD. Statistical analysis was performed by one-way ANOVA.

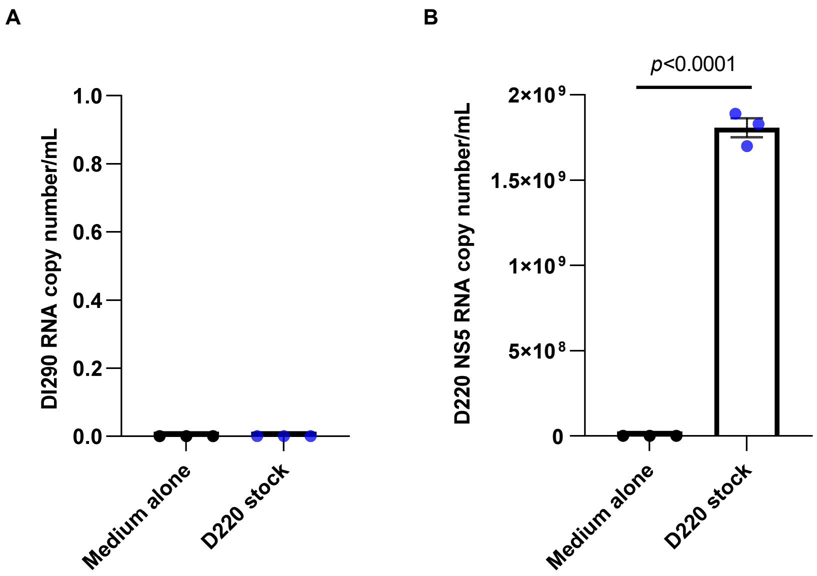

Figure S8. **Levels of DI290 in D220 virus stocks.** (A and B) D220 virus stock was treated with Trition X-100 (final concentration of 0.5%) and RNasin RNase inhibitor. The levels of DI290 and D220 RNA were measure by RT-qPCR using primers targeting the DI290 and D220 NS5 regions, respectively (n=3). Data are shown as the mean ± SD. Statistical analysis was performed by student's *t*-test.

Supplementary Tables

Table S1. RNA-seq results of DEGs in *Ifnar*$^{-/-}$ mice treated with either empty LNP or LNP-DI290.

Table S2. RNA-seq results of DEGs in human MDMs treated with either empty LNP or LNP-DI290.

Table S3. RNA-seq results of DEGs in human MDMs treated with either buffer or DENV DIP.

Table S4. RNA-seq results of DEGs in C57BL/6J mice treated with either empty LNP or LNP-DI290.
